# CNPY2 protects against ER stress and is expressed by corticostriatal neurons together with CTIP2 in a mouse model of Huntington’s disease

**DOI:** 10.3389/fnmol.2024.1473058

**Published:** 2024-09-18

**Authors:** Miriana Scordino, Polina Stepanova, Vignesh Srinivasan, Dan Duc Pham, Ove Eriksson, Maciej Lalowski, Giuseppa Mudò, Valentina Di Liberto, Laura Korhonen, Merja H. Voutilainen, Dan Lindholm

**Affiliations:** ^1^Department of Biochemistry and Developmental Biology, Medical Faculty, University of Helsinki, Helsinki, Finland; ^2^Biomedicum-2, Minerva Foundation Institute for Medical Research, Helsinki, Finland; ^3^Department of Biomedicine, Neuroscience, and Advanced Diagnostic (BiND), University of Palermo, Palermo, Italy; ^4^Faculty of Pharmacy, University of Helsinki, Helsinki, Finland; ^5^Meilahti Clinical Proteomics Core Facility, HiLIFE, University of Helsinki, Helsinki, Finland; ^6^Department of Gene Expression, Institute of Molecular Biology and Biochemistry, Adam Mickiewicz University, Poznań, Poland; ^7^Department of Child and Adolescent Psychiatry, Linköping University, Linköping, Sweden; ^8^Department of Biomedical and Clinical Sciences, Linköping University, Linköping, Sweden

**Keywords:** UPR, ER stress, CNPY2, CTIP2, nerve cell viability, Huntington’s disease

## Abstract

Canopy Homolog 2 (CNPY2) is an endoplasmic reticulum (ER) localized protein belonging to the CNPY gene family. We show here that CNPY2 is protective against ER stress induced by tunicamycin in neuronal cells. Overexpression of CNPY2 enhanced, while downregulation of CNPY2 using shRNA expression, reduced the viability of neuroblastoma cells after tunicamycin. Likewise, recombinant CNPY2 increased survival of cortical neurons in culture after ER stress. CNPY2 reduced the activating transcription factor 6 (ATF6) branch of ER stress and decreased the expression of CCAT/Enhancer-Binding Protein Homologous Protein (CHOP) involved in cell death. Immunostaining using mouse brain sections revealed that CNPY2 is expressed by cortical and striatal neurons and is co-expressed with the transcription factor, COUPTF-interacting protein 2 (CTIP2). In transgenic N171-82Q mice, as a model for Huntington’s disease (HD), the number of CNPY2-immunopositive neurons was increased in the cortex together with CTIP2. In the striatum, however, the number of CNPY2 decreased at 19 weeks of age, representing a late-stage of pathology. Striatal cells in culture were shown to be more susceptible to ER stress after downregulation of CNPY2. These results demonstrate that CNPY2 is expressed by corticostriatal neurons involved in the regulation of movement. CNPY2 enhances neuronal survival by reducing ER stress and is a promising factor to consider in HD and possibly in other brain diseases.

## Introduction

Alterations in protein homeostasis and cell signaling pathways accompany many human neurological diseases and are associated with organelle dysfunctions and cell stress, exemplified by endoplasmic reticulum (ER) stress (Lindholm et al., 2006; Hetz and Saxena, 2017). The contributions of the different pathways to the disease development are however, not fully understood. Recently the regulation of the unfolded protein response (UPR) and ER stress in neurodegenerative disorders has received increased attention (Walter and Ron, 2011; Wang and Kaufman, 2012; Hetz et al., 2015). The UPR is an adaptive mechanism in the cell, which reduces the harmful effects of misfolded or unfolded proteins accumulating in the ER. During the UPR specific signaling cascades are activated, including the inositol-requiring enzyme-1α (IRE1α), the PRK-like ER protein kinase/pancreatic eIF2α kinase (PERK), and the activating transcription factor-6 (ATF6) (Walter and Ron, 2011; Hetz et al., 2015). An increasing number of studies have shown that ER associated factors and cellular chaperons can modulate UPR signaling, to positively influence cell viability (Lindahl et al., 2017; Sampieri et al., 2019).

Canopy Homolog 2 (CNPY2) belongs to the family of CNPY proteins that are mainly localized to the ER. These proteins have different functions including effects on cell signaling, protein trafficking, lipid metabolism, cell adhesion and host defense (Schildknegt et al., 2019). We have previously cloned CNPY2 having a saposin-like structure with a HDEL sequence at the carboxyterminal end (Bornhauser et al., 2003). Subsequently, it was shown that CNPY2 plays a role in neurite outgrowth, in cell migration and in the regulation of low-density lipoprotein receptor (Bornhauser and Lindholm, 2005; Do et al., 2012). CNPY2 is also known to influence cell signaling in the context of tumor cell growth (Yan et al., 2016; Ito et al., 2018; Kakehashi et al., 2021), and the protein also affects angiogenesis and promotes cardiac regeneration (Guo et al., 2015a,b; Yin et al., 2019). Herein we have investigated the function of CNPY2 in the ER stress regulation in neuronal cells. We have further investigated its expression in the mouse brain and in N171-82Q mice as a model for Huntington’s disease (HD) we studied before (Stepanova et al., 2023). Data showed that CNPY2 was able to increase the viability of neuronal cells after ER stress by modulating UPR signaling. Particularly, the expression of the CCAT/Enhancer-Binding Protein Homologous Protein (CHOP) associated with an enhanced cell demise was reduced by CNPY2.

Immunostaining revealed that CNPY2 is present in cortical and striatal neurons and co-expressed with the COUPTF-interacting protein 2 (CTIP2), also called as B-cell lymphoma/leukemia 11 B (BCL11B) (Avram et al., 2000; Arlotta et al., 2008; Desplats et al., 2008). CNPY2 was increased in cortical neurons in the N171-82Q mouse brain together with CTIP2. In the striatum, the number of CNPY2 immunopositive neurons was reduced in N171-82Q mice at 19 weeks of age, representing a late phase of pathology (Schilling et al., 2001; Ferrante, 2009; Stepanova et al., 2023). Together these results show that CNPY2 is expressed by neurons in the corticostriatal circuitry regulating body movements. Changes in the corticostriatal circuitry and degeneration of striatal neurons play a crucial role in the pathophysiology of HD. Future studies including gene deleted mice are warranted to reveal the significance of CNPY2 in disease models of HD.

## Methods

### Animal model

In this study we used brain tissue from transgenic N171-82Q mice that express mutant N-terminal fragment of huntingtin (Htt) with 82 polyglutamine repeats under the mouse prion protein promoter (Schilling et al., 2001). The transgene expression is restricted to neurons in the N171-82Q mice and the mice show behavioral symptoms at 2–3 months of age with a lifespan of about 16–22 weeks (Ferrante, 2009; Ramaswamy et al., 2009; Stepanova et al., 2023). Brain samples from the striatum and cortex were prepared from the N171-82Q mice aged 10 to 20 weeks, together with age-matched brain tissue from control C57BL/6JRccHsd mice (Arlotta et al., 2008). The brains were frozen and analyzed by immunolabeling as described below. To study the protein expression of CNPY2, the parietal cortex and striatum were dissected and stored frozen for subsequent immunoblotting experiments. The mice were handled in the animal facility under strict conditions of care and surveillance. Experiments were approved by the ethical committee with the license numbers, ESAVI/8897/04.10.07/2017, and ESAVI/27113/2020.

### Immunostaining

Immunohistochemistry was done as described before (Stepanova et al., 2023). The number of animals used for these experiments are given in Supplementary Table 1. The N171-82Q mice and wildtype control mice were anaesthetized and perfused transcardially with PBS followed by 4% paraformaldehyde (PFA) in PBS. Brains were collected, post-fixed overnight in 4% PFA, and stored in 4% PFA for paraffin embedding or in 4% PFA followed by 30% sucrose for free-floating slices. The brains were cut into 10–30 μm-thick slices for immunohistochemical analysis. Paraffin slices (10 μm) were deparaffinized using xylene, washed by decreasing concentrations of ethanol followed by deionized water. Antigen retrieval was done by heating sections in 10 mM citrate buffer pH 6.0, in 0.05% Tween-20, for 10 min followed by rinsing with PBS, and blocking using 1% BSA, 0.2% Triton-X-100 in PBS for 1 h. Primary antibodies were added and included: rabbit polyclonal anti-CNPY2 (diluted 1:250, 14,635-1-AP, Proteintech), rat anti-CTIP2 (diluted 1:200, ab18465, Abcam), mouse monoclonal anti-NeuN (1:300, #MAB377, Chemicon), and rabbit anti-CNPase (1:300, AB9342, Merck). For double immunostainings, the specific antibodies were added in combination. Following incubation overnight at 4°C, the slices were washed with PBS-T buffer and incubated further with fluorescence secondary antibodies, including donkey anti-rabbit, goat anti-mouse and anti-rat antibodies (diluted 1:400; Vector Laboratories, Burlingame, CA) for 1 h at room temperature. The slices were washed three times with PBS-T buffer, and DAPI solution (1:1000 in PBS) was added for 5 min to visualize nuclei. Following additional washings, the slices were coverslipped with Dako fluorescence mounting medium and kept at 4°C in the dark. The free-floating slices (30 μm) were washed with PBS, and the procedure was then similar to the above but without the antigen retrieval step.

Slices were mounted onto slides, dehydrated, cleared in xylene, and mounted with DPX (Biolab). Images were generated using 3DHISTECH Panoramic 250 FLASH II digital slide scanner at Genome Biology Unit, HiLIFE and the Faculty of Medicine, University of Helsinki. Control samples were without primary antibodies.

To count the number of CNPY2 and CTIP2 immunopositive cells we employed ImageJ software (ImageJ, University of Wisconsin-Madison, USA). Fluorescence images of the scans were captured with a 60x magnification from cortical layers 5/6 and from upper striatum in 19–20 weeks old control and N171-82Q mice. The total number of brains used for cell counts in the immunohistochemical analysis amounted to 8 control and 8 N171-82Q mice. The number of immunolabeled cells was quantified from 3 square images in each of the brain areas of the different controls and N171-82Q mice by two independent researchers blind to the experimental groups.

### Plasmids and reagents

Plasmids for CNPY2 were generated by cloning of CNPY2 from cDNA of rat brain and further inserted it into expression vectors with GFP or dsRed tags for expression in cells (Bornhauser et al., 2003; Do et al., 2012). CNPY2-Myc-DDK plasmid was obtained from OriGene Technologies, Inc. (Rockville, MD, USA, plasmid RC209441). To downregulate CNPY2 in cells, we used the short hairpin (sh)-RNA CNPY2 construct (SABiosciences, Frederick, MD, USA) as described before (Do et al., 2012). Recombinant human CNPY2 was from Origene Technologies Inc. (no. TP309441), and 100 ng/mL protein was added to neuronal cultures as described below.

### Cell cultures

In this work, different cells were used to study the role of CNPY2 in cell signaling. Supplementary Figure S1 shows images of the different cells in culture.

### Human neuroblastoma SH-SY5Y cells

**Figure 1:**
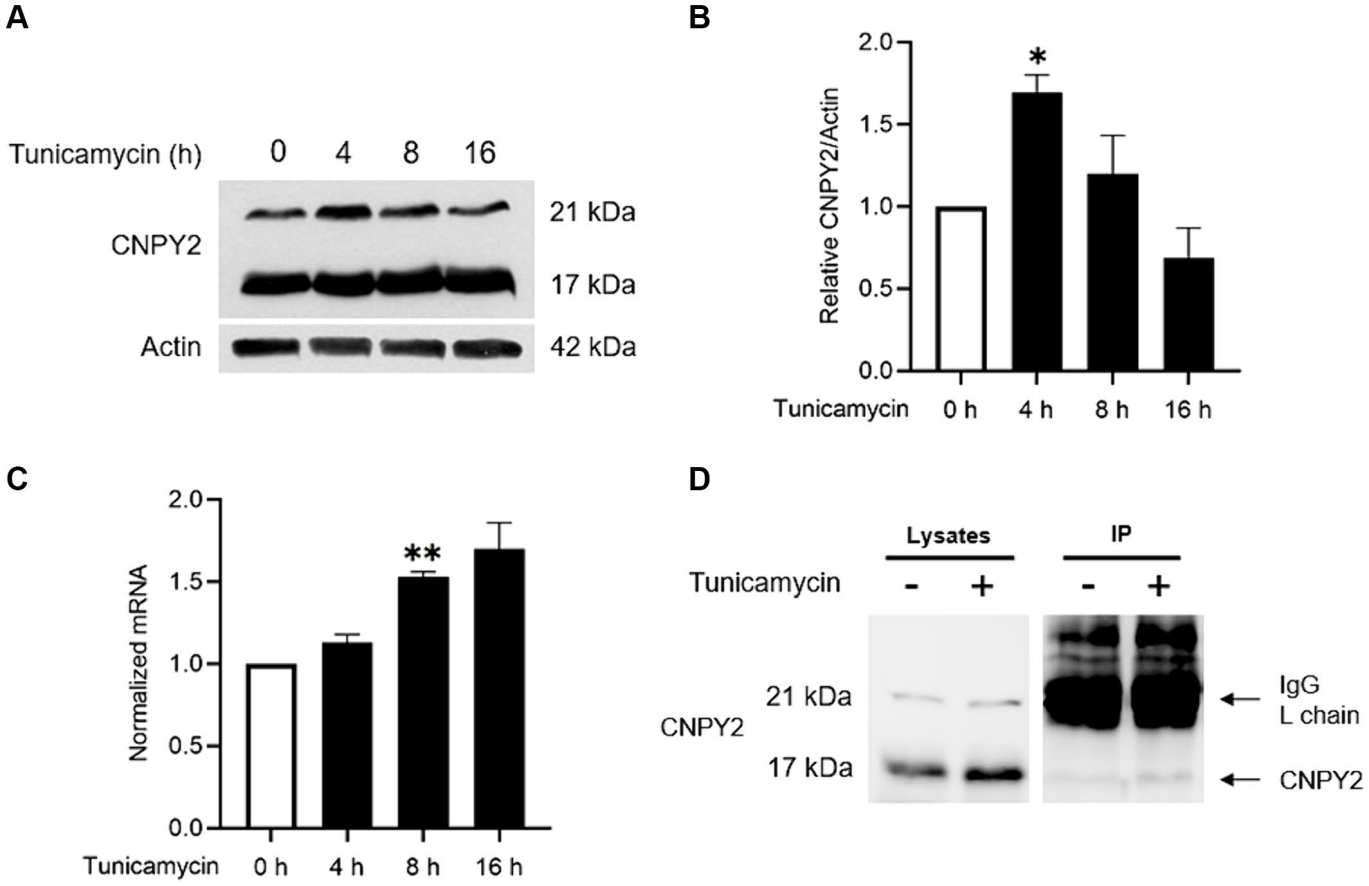
Tunicamycin increases CNPY2 expression and protein levels in neuronal cells. SH-SY5Y cells were treated with 2.5 μg/mL Tunicamycin (Tun) for various times and the levels and expression of CNPY2 were determined in cells by immunoblotting or quantitative PCR (qPCR) respectively. Cell medium was analyzed separately using immunoprecipitation (IP) as described in methods. **(A,B)** Immunoblotting was done using anti-CNPY2 antibodies and β-actin as control. Left immunoblots. CNPY2 is present as 21 kDa and 17 kDa size bands. The level of 21 kDa band was used for quantification and β-actin. Right quantification. Values are means ± SD, *n* = 4. ANOVA. ^*^p < 0.05 for Tun 4 h vs. untreated control. **(C)** Quantitative PCR for CNPY2 using β-actin as control. There was an increase in CNPY2 expression after Tun treatment. Values are means ± SD, *n* = 4. ^**^p < 0.01 for Tun vs. control. **(D)** Cell media were collected from control and Tun-treated cells at 7 h post-treatment. IP was done using anti-CNPY2 antibody followed by immunoblotting. Left, cell lysates. Right, IP showing the presence of 17 kDa CNPY2 in the cell medium at h post-treatment. The IgG light chain in the IP is shown by the upper arrow.

Human neuroblastoma cells were cultured at 37°C in 5% CO_2_ in 10% fetal bovine serum (Thermo Fisher Scientific, Waltham, MA, USA) using Dulbecco’s Modified Eagle Medium (DMEM) containing 100 mM L-glutamine and 100 mM penicillin–streptomycin (all from Thermo Fisher Scientific). Cells were treated with tunicamycin for different periods of time (Figure 1), and analyzed for the expression of CNPY2 using RT-PCR or immunoblotting as described below. To study the effects of CNPY2 on ER signaling pathways the cells were transfected with control GFP or CNPY2 expressing plasmids using transfectin or polyethylenimine (PEI) 25.000 (Polysciences Warrington, PA, USA) based on the manufacturer’s instructions. Briefly, PEI was prepared at a stock concentration of 1 mg/mL and combined with DNA at a ratio of 3:1 and added to cells. Cells were further treated with tunicamycin and the levels of protein expressed were determined by immunoblotting.

**Figure 2:**
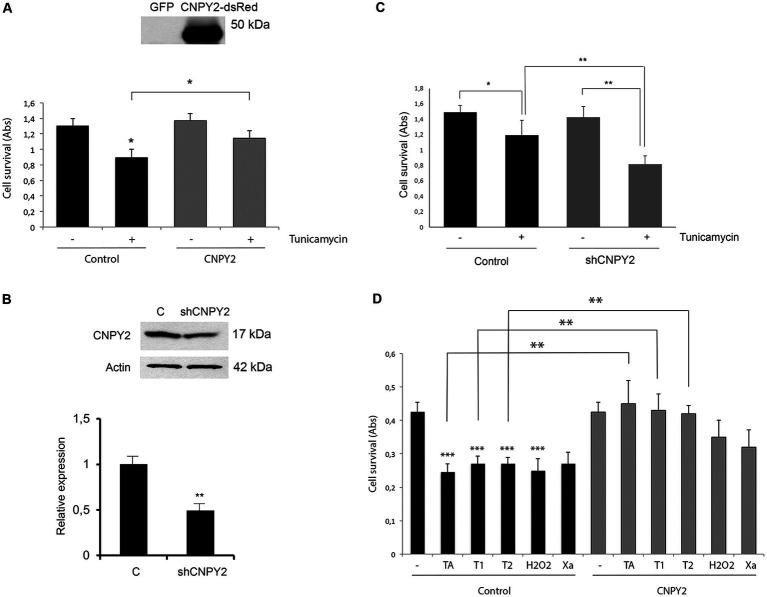
CNPY2 expression protects against ER stress in neuronal cells. Mouse N2A neuroblastoma **(A–C)** and human SH-SY5Y cells **(D)** were used for experiments. **(A)** N2A cells were cultured in 96 well plates and transfected with 4 ug EGFP control, or CNPY2- dsRed expressing plasmid. Cells were further treated with 2.5 μg/mL Tunicamycin (Tun) for 24 h and cell viability was determined by the MTT assay as described in the methods. For the experiment 8 wells were used for each treatment and the experiments were repeated three times. Note the reduced cell viability after Tun treatment that was increased by CNPY2 expression. The insert shows the expression level of CNPY2-dsRed in transfected cells. Molecular weight marker is 50 kDa. Values are means ± SD, *n* = 4. ^*^p < 0.05 for Tun vs. C, and for CNPY2 + Tun vs. Tun. **(B)** N2A cells were transfected with 4 μg shRNA-CNPY2 expressing plasmid as described in the methods. Control cells were transfected with scrambled plasmid. The 17 kDa CNPY2 level was decreased by about half using the sh-RNA. A typical experiment is shown and was repeated with similar results. Values are means ± SD, *n* = 3. ^*^p < 0.01 for Tun vs. control. **(C)** N2A cells were treated with 2.5 μg/mL Tun for 16 h. Downregulation of CNPY2 using shRNA reduced the cell viability caused by Tun treatment. Values are means ± SD, *n* = 4. ^*^p < 0.05 for Tun vs. C. ^**^p < 0.01 for shCNPY2 + Tun vs. shRNA-CNPY, and for shRNA-CNPY2 + Tun vs. C + Tun. **(D)** Human SH-SY5Y neuroblastoma cells with stable overexpression of CNPY2 were generated as described in methods. Cells were then treated for 24 h with different compounds as indicated and cell viability was determined using 8 wells for each treatment, and the experiments were repeated three times. There was a significant increase in cell viability in CNPY2 overexpressing cells compared with controls after treatments with 1 μM thapsigargin (TA), 2.5 μg/mL (T1) or with 5 μg/mL (T2) tunicamycin. Notably, CNPY2 did not significantly protect against cell loss induced by 400 μM H_2_O_2_ or by using Xantine (Xa)/Xantine oxidase causing oxidative stress. Values are means ± SD, *n* = 3. ^**^p < 0.01 for Tun vs. untreated control for all treatments, and for CNPY2 overexpressing cells with TA, T1, or T2 vs., controls with TA, T1, or T2.

In order to obtain cells with stable expression of CNPY2, human SH-SY5Y cells were transfected with CNPY2 Gateway™ cloned to pES-NTAP-Puro vector (Srinivasan et al., 2020). Puromycin was added and resistant clones were selected and cultured further for the experiments. Control SH-SY5Y cells were transfected with pES-NTAP-Puro vector and treated similarly. For the experiments, control and CNPY2 overexpressing cells were treated with tunicamycin or thapsigargin (both from Sigma-Aldrich, St. Louis, MO, USA) as described in Figure 2 and analyzed for cell viability using MTT (see below). To study the role of oxidative stress, the cells were either treated with 400 μM H_2_O_2_ for 24 h, or alternatively with 50 mM xanthine together with 50 mU/mL xanthine oxidase (both from Sigma-Aldrich) as described before (Kairisalo et al., 2011). Cell viability was determined by MTT as described below.

**Figure 3:**
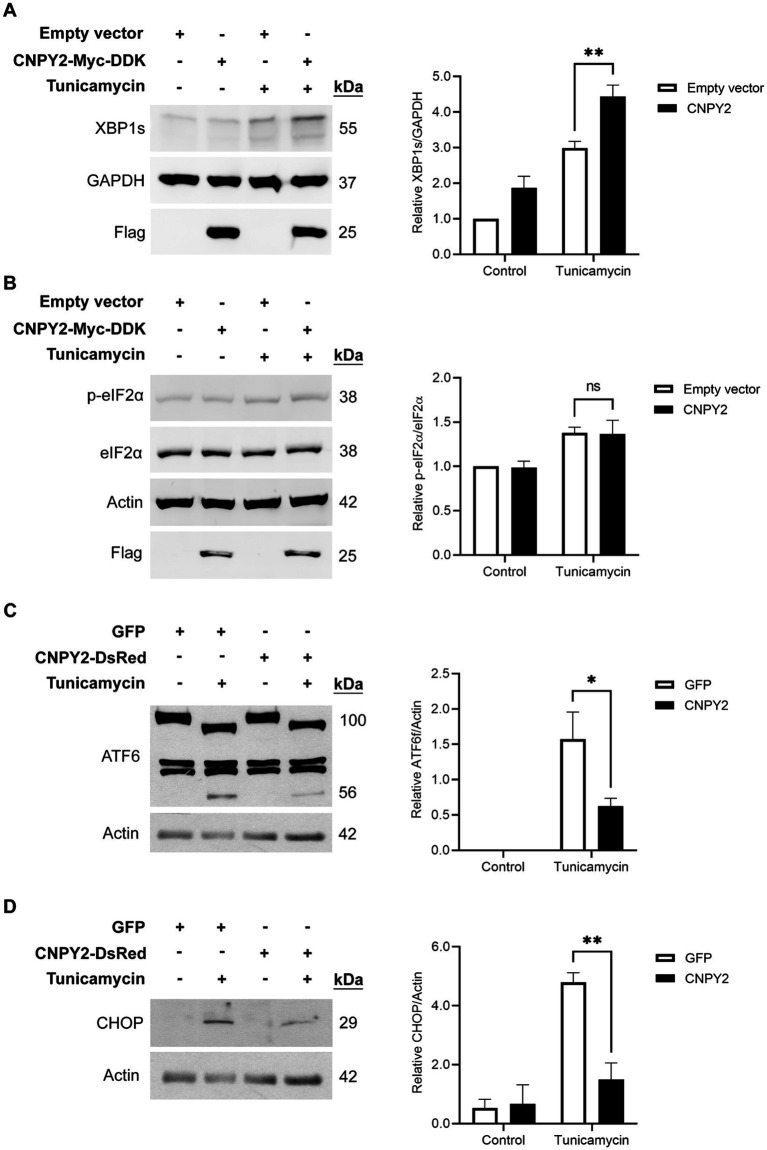
CNPY2 regulates UPR signaling pathways. SH-SY5Y cells were transfected with control plasmid or with CNPY2-Myc-DDK **(A,B)** or CNPY2-dsRed **(C,D)** expressing plasmid for 24 h and cells were further treated with 2.5 μg/mL tunicamycin (Tun) for different times followed by immunoblotting as described above using β-actin (42 kDa) or GAPHD (37 kDa) as controls. Left, immunoblots, Right, quantification. Values are means ± SD, *n* = 4–5. ^*^p < 0.05 or ^**^p < 0.01 for CNPY2 + Tun vs. Tun. **(A,B)** Tun was added for 6 h. The level of spliced XBP1 (XBP1s) was increased by Tun and was further elevated by CNPY2 expression **(A)**. The level of phosphorylated eIF2α was increased by Tun but was not significantly affected by CNPY2 expression **(B)**. **(C)** Tun was added for 16 h to study ATF6α signaling. The processing of ATF6α was induced by Tun as shown by the decrease in full length protein (100 kDa) and the appearance of the cleaved ATF6α fragment (56 kDa) in the immunoblots. CNPY2 expression reduced this ATF6α processing. **(D)** Tun was added for 24 h. The transcription factor CHOP (27 kDa) was induced by Tun and was decreased by CNPY2 expression.

### Mouse neuroblastoma Neuro2A cells

Mouse neuroblastoma cells were cultured at 37°C in 5% CO_2_ in 10% fetal bovine serum, using DMEMcand 100 mM L-glutamine and 100 mM penicillin–streptomycin. Cells were transfected with control GFP or dsRed CNPY2 expressing plasmids as described above. To downregulate CNPY2, the cells were transfected with sh-RNA against CNPY2 (Do et al., 2012). Control cells received scrambled plasmid. To induce ER stress, the control and the CNPY2 modulated cells were then treated with tunicamycin as described in Figure 2, and cell viability was determined by MTT.

### Striatal cells

Striatal cells derived from mice and immortalized with a temperature-sensitive large T antigen were cultured as described before (Kannike et al., 2014). Striatal cells expressing 7-polyglutamine (7Q) Htt protein were used for the experiments and grown at 33°C in Dulbecco’s Modified Eagle’s medium (DMEM, Invitrogen) supplemented with 10% fetal bovine serum. To induce neuronal differentiation, cells were incubated at 37°C for 1 day. Cells were transfected with shRNA-CNPY2 or control plasmid as above and treated with tunicamycin followed by immunoblotting and analyses of cell viability.

### Primary neuron cultures

Neurons were prepared from embryonic E17-old rat brains and plated onto poly-L-ornithine (Sigma-Aldrich) coated cultured dishes at a density of 0.25 × 10^6^ cells per well (Li et al., 2015). Cells were cultivated at 37°C in 5% CO_2_ in Neurobasal medium supplemented with 50x B27, 25 mM glutamine (all from Gibco) and 100 mM penicillin–streptomycin for 7 days as described before (Srinivasan et al., 2020). The cultures contained less than 5% GFAP positive astrocytes. Neurons were treated or not with tunicamycin for 24 h in the absence or presence of 100 ng/mL recombinant CNPY2, and cell viability was determined using MTT.

### Cell viability assay

Cell viability was examined using the tetrazolium dye, 3-(4,5-dimethylthiazol-2-yl)-2,5-diphenyltetrazolium bromide (MTT, Sigma-Aldrich) that was added to cells for 2 h. The insoluble formazan substrate formed was reconstituted using 0.1 M HCl-isopropanol and absorbance was measured at 560 nm. The values correspond to the relative number of surviving cells present in the cultures.

### Western blot analyses

Immunoblotting was done as described previously (Srinivasan et al., 2020). Cells in culture were washed twice with ice-cold PBS and lysed in RIPA buffer (50 mM Tris–HCl, pH 7.4, 1% Triton-X-100, 0.5% sodium deoxycholate, 1% SDS, and 150 mM NaCl) supplemented with protease inhibitors (Roche, Penzberg, Germany) and phosphatase inhibitors (Roche). To study the levels of CNPY2, tissues taken from the parietal cortex and striatum were homogenized and processed similarly as described above.

The lysates were sonicated twice for 4 s and centrifuged at 10.000 g for 15 min at 4°C, and the protein concentrations were determined by the Pierce BCA Protein Assay Kit (Thermo Fisher Scientific). Equal amounts of protein were separated by SDS-PAGE gel followed by transfer onto a nitrocellulose membrane (GE Healthcare, Chicago, IL, USA). The membranes were blocked for 1 h in 5% skimmed milk or bovine serum albumin in TBS-T buffer (50 mM Tris–HCl pH 7.5, 150 mM NaCl, 0.1% Tween 20) and incubated with primary antibodies overnight at 4°C with gentle agitation. Antibodies included: anti-CNPY2 (diluted 1:4000, 14,635-1-AP, Proteintech, Manchester, UK), anti-ATF6α (1:1000, 24,169-1-AP, Proteintech; or ALX-804-381-C100, Enzo Life Sciences, Farmingdale, NY, USA), anti-CHOP (1:2000, 2,895, Cell Signaling Technology (CST), Danvers, MA, USA; sc-7351, Santa Cruz Biotechnology, Dallas, TX, USA), anti-BiP (1:1000, ab21685 Abcam), anti-eIF2α (1:1000, 5,324, CST), anti-phospho-eIF2α (1:1000, 3,597, CST), anti-XBP1 (1:2000, D2C1F, CST). Anti-ß-actin (1:1000, Sigma, A2066, Sigma-Aldrich) or anti-GAPDH (MAB374, Millipore, Burlington, MA, USA) were used as controls in the immunoblots, and anti-GFP (1:2000, Sigma-Aldrich) or anti-Flag (1,2000, Sigma-Aldrich) to monitor plasmid expression. Membranes were washed and incubated with horseradish peroxidase-conjugated secondary antibodies (1,2,500, Jackson ImmunoResearch Europe, Cambridge, UK) for 1 h at room temperature. Protein signals were detected using enhanced chemiluminescence substrate (Pierce Protein Biology, Thermo Fisher Scientific). Immunoblots were quantified with ImageJ (NIH) quantification software.

### Immunoprecipitation

Culture media from SH-SY5Y cells were collected from untreated or treated cells at 7 h after tunicamycin and subjected to immunoprecipitation by interacting the medium with Protein A/G agarose beads (Sigma-Aldrich) for 1 h at 4°C. Pre-cleared suspension was incubated with 2 μg anti-CNPY2 at 4°C overnight, agarose beads were added, and the slurry was rotated at 4°C for 2–3 h. The beads were centrifuged at 9000 g for 1 min at 4°C, washed three times with buffer for 5 min on a rotator at 4°C. The immunoprecipitate was resuspended in 2x Laemmli buffer and heated at 95°C for 5 min. The samples were further centrifuged for 3 min at 10.000 g, and the supernatant was collected and analyzed by immunoblotting.

### Quantitative PCR

RNA was extracted from SH-SY5Y cells using the RNeasy mini kit (Qiagen). cDNA was synthesized with SuperScript VILO (Thermo Fisher Scientific) following instructions by the manufacturer. Real time quantitative PCR (qPCR) was performed using the Light Cycler 480 II instrument (Roche Diagnostics, Basel, Switzerland) using specific primers and SYBR Green Master Mix (Roche). Reactions were carried out at 95°C for 10 min followed by at 95°C for 15 s, 60°C for 20 s, and 72°C for 10 s using 40 cycles. Each sample was run in triplicates on a 96-well plate, and water was used as a negative control. Expression levels were calculated based upon the 2^–ΔΔCT^ method using GAPDH as control. The following primer sequences were used: CNPY2, forward, 5′- TGGGATCTTTCCGGATCA-3′, and reverse, 5’-CTCTGAGCGGGCATAAGG-3′; GAPDH, forward, 5′-TTCGTCATGGGTGTGAACCA-3′, and reverse, 5′-CTGTGGTCATGAGTCCTTCCA-3′.

### Statistical analysis

The normal distribution of data was assessed via the Shapiro–Wilk test. Statistical evaluations were performed using Student’s *t*-test or one-way/two-way ANOVA followed by a Bonferroni *post hoc* test depending on the experimental design. Values are presented as means ± SD. *p* < 0.05 was considered statistically significant. Statistical analysis and graph design were done using GraphPad PRISM software version 7.02. (GraphPad, CA, USA).

## Results

### CNPY2 is increased by tunicamycin and is present in the cell medium

CNPY2 is an ER-localized protein, and we therefore investigated whether CNPY2 itself could be regulated by the UPR. For this, cultured human SH-SY5Y neuroblastoma cells were treated with tunicamycin for different times followed by immunoblotting. Data showed that the protein levels of CNPY2 were significantly increased by tunicamycin with a peak at 4 h (Figures 1A,B), together with an upregulation of CNPY2-mRNA levels as shown by qPCR (Figure 1C). The increase in protein levels of CNPY2 was not always correlated to the corresponding mRNA levels suggesting some post-transcriptional alterations in CNPY2 that warrant further studies. In the immunoblots, two bands of CNPY2 were detected with the molecular weights of about 21 kDa and 17 kDa (Figure 1A). The lower band likely represents the mature form of CNPY2 following the removal of the signal sequence. We thus investigated whether the 17 kDa CNPY2 band is to be found in the medium of SH-SY5Y cells, by using immunoprecipitation to enrich for CNPY2 (Figure 1D). Results showed that the 17 kDa CNPY2 is present in the cell medium in accordance with a secretion of the protein (Figure 1D). The intensity of the 17 kDa band seemed to be slightly increased by tunicamycin at 7 h after tunicamycin, but this was not quantified further in these experiments. Apart from tunicamycin we observed that thapsigargin was able to increase the CNPY2 protein levels in SH-SY5Y cells (Supplementary Figure 2). Altogether, these results demonstrate that CNPY2 expression and protein levels are increased by tunicamycin in neuronal cells, and that 17 kDa CNPY2 is present in the cell medium.

### CNPY2 reduces ER stress mediated cell demise and the induction of CHOP

We next studied whether the modulation of the expression levels of CNPY2 can influence cell viability after inducing ER stress by tunicamycin. In these experiments mouse neuroblastoma N2A cells were transfected with CNPY2-dsRed overexpressing construct or by using short hairpin (sh) RNA to reduce CNPY2 levels. Cells were subsequently treated with 2.5 μg/mL tunicamycin for 24 h followed by analyses of cell viability. Data showed that tunicamycin decreased cell viability by about one-third at 24 h and this was counteracted by the overexpression of CNPY2 (Figure 2A). In contrast, downregulation of CNPY2 using the shRNA construct (Do et al., 2012) rendered the N2A cells more prone to loss of cell viability induced by tunicamycin (Figures 2B,C).

To substantiate the data, we also studied cell clones of SH-SY5Y neuroblastoma cells stably overexpressing CNPY2. In control SH-SY5Y cells, tunicamycin or thapsigargin reduced cell viability to about half observed with untreated cells (Figure 2D). In cells overexpressing CNPY2, the effects of tunicamycin and thapsigargin to reduce cell viability were significantly lower (Figure 2D). To study whether CNPY2 may also act against other types of stress we investigated oxidative stress induced by the addition of H_2_O_2_ or by using the combination of xanthine and xanthine oxidase (Srinivasan et al., 2020). However, CNPY2 did not significantly protect SH-SY5Y cells against oxidative stress under these conditions (Figure 2D). Together these findings demonstrate that CNPY2 overexpression evokes cell protection against ER stress in neuroblastoma cells.

To investigate the underlying mechanisms, we studied ER stress-linked signaling pathways in SH-SY5Y cells transfected with either control or CNPY2 plasmids. Expression of CNPY2 was found to increase the levels of spliced XBP1 (XBP1s) induced by tunicamycin in the SH-SY5Y cells (Figure 3A). XBP1s is linked to the activation of IRE1α during ER stress, indicating that CNPY2 can activate this UPR pathway. In contrast, there was no significant change in the phosphorylation of eIF2α by CNPY2 (Figure 3B), suggesting that the PERK pathway was not significantly affected by CNPY2 expression under these conditions. ATF6 is the third arm of UPR and the protein is processed by the action of the Site-1 protease (and Site-2 protease causing the release of the amino-terminal part of the molecule; Haze et al., 1999; Ye et al., 2000). Tunicamycin induced the processing of the full length ATF6α to a 100 kDa protein type and a lower 56 kDa type, representing the cleaved, transcriptionally active form of ATF6α, in control SH-SY5Y cells (Figure 3C). In CNPY2 overexpressing cells, the processing of ATF6α was significantly reduced compared with control GFP-expressing cells (Figure 3C), suggesting a lower activation of the ATF6 pathway. To study whether CNPY2 overexpression affects downstream genes, we studied the CCAT/Enhancer-Binding Protein Homologous Protein (CHOP) that is associated with cell death following ER stress (Marciniak et al., 2004; Li et al., 2015). Data showed that CNPY2 overexpression repressed the increase in CHOP induced by 24 h treatment with tunicamycin in the SH-SY5Y cells (Figure 3D). These results demonstrate that CNPY2 can mitigate the ATF6 pathway and reduce CHOP levels in the neuroblastoma cells. To study the mode of cell death induced by tunicamycin we investigated the activation of caspase-3 using immunblotting. Data obtained revealed that caspase-3 is cleaved (17 kDa protein) by tunicamycin in the SH-SY5Y cells and this was reduced by overexpression of CNPY2 (Supplementary Figure 3). The precise mechanisms by which CNPY2 can counteract CHOP activation and subsequent cell death involving caspase-3 warrant further investigations.

### Recombinant CNPY2 increases the viability of primary neurons after ER stress

**Figure 4:**
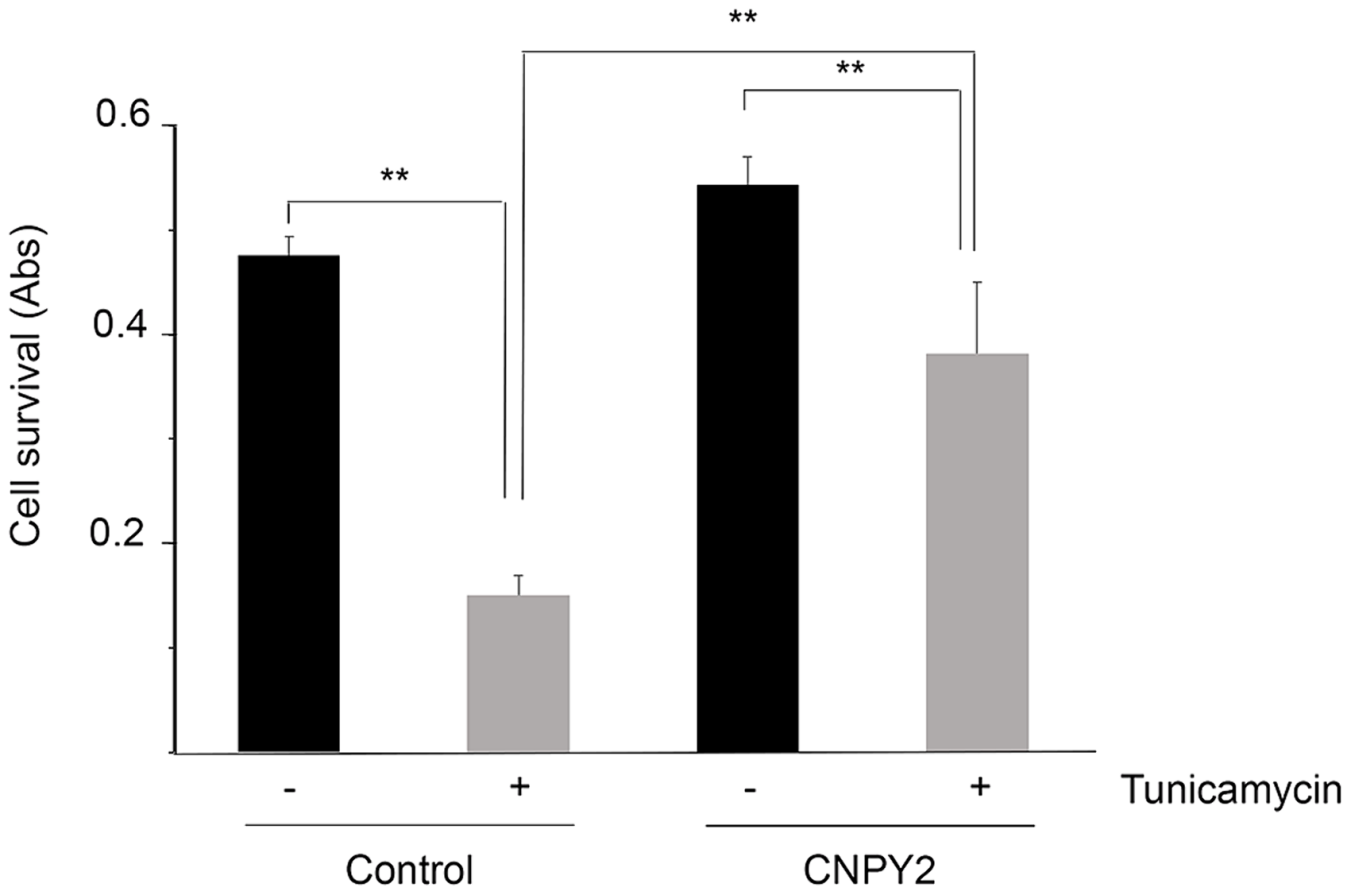
Recombinant CNPY2 increases the survival of primary cortical neurons after ER stress. Neurons were prepared from brain cortices of E18 old rats and grown in culture for 7 days as described in Methods. Neurons (0.25 × 10^6^ cells) in 96 well plates were then treated with 2.5 μg/mL tunicamycin (Tun) for 24 h in the absence or presence of 100 ng/mL recombinant CNPY2. For the experiment, eight wells were used for each treatment that was done in triplicates. Cell viability was determined by MTT assay as described in Methods. Tun decreased cell viability of the neurons and this was counteracted by the addition of CNPY2. Values are means ± SD, *n* = 4. ^**^p < 0.01 for T vs. C, and for CNPY2 + T vs. T.

The experiments above demonstrate the protective role of CNPY2 against ER stress using neuronal cell lines. To study whether CNPY2 is equally effective in primary neurons, we prepared neurons from embryonic rat brain and cultured the cells for 7 days as described in “Methods.” Subsequently the cells were treated with tunicamycin for 24 h to induce ER stress in the absence or presence of 100 ng/mL recombinant CNPY2. Data obtained showed that there was a significant increase in cell viability of cortical neurons in the presence of recombinant CNPY2 as estimated using the MTT assay (Figure 4). These results demonstrate that CNPY2 can increase the survival of cortical neurons under conditions of ER stress and that recombinant CNPY2 acting from outside of the cell is also active in this paradigm.

### CNPY2 is expressed by cortical and striatal neurons together with CTIP2 and is altered in HD N171-82Q mice

**Figure 5:**
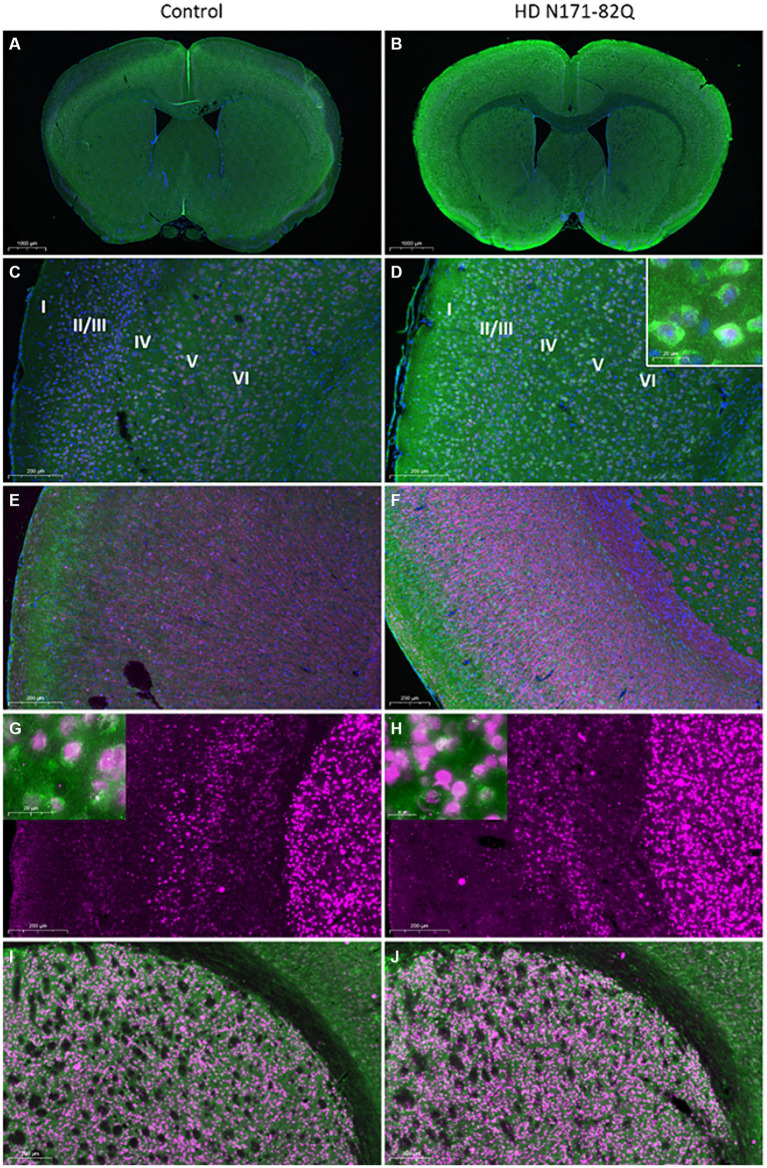
Immunohistochemistry of CNPY2 and CTIP2 in mouse brain cortex and striatum. The number of immunopositive CNPY2 and CTIP2 cells in the cortex and in the striatum was determined by immunostaining in 16-week-old **(A–F)** or 19-week-old **(G–J)** control and HD N171-82Q mice. **(A–F).** Immunostaining of CNPY2 was performed on paraffin slices from 16-week-old mice as described in Methods in conjunction with anti-NeuN antibodies as a marker for neurons. Left, Control female mice **(A,C,E)**. Right similar HD N171-82Q female mice **(B,D,F)**. **(A,B)** CNPY2 positive cells are shown in green color, NeuN positive cells are red/violet, and nuclei are stained by DAPI (blue color). Note an increased immunolabeling of CNPY2 in the N171-82Q mice. **(C,D)** Cortical layers are here labeled I to VI. The insert in (D) shows NeuN immunopositive nuclei with CNPY2 in the cytosol. **(E,F)** CNPY2 (green) immunolabeled cells are present in cortical layer 2/3 and 5/6 and are increased in the N171-82Q mice. Myelin immunolabeled by the CNPase antibody is in red and encircles the axons of the cortical neurons. Scale bars: **(A,B)** 1,000 μm; **(C–F)** 200 μm. **(G-J).** Immunostaining of CTIP2 and CNPY2 in 19-week-old mouse brain. Free-floating slices from control **(G,I)** and N171-82Q mouse brain **(H,J)** were immunostained as described above in conjunction with an antibody against the transcription factor, CTIP2. **(G,H)** Immunopositive CTIP2 cells (red color) in cortical layer 5 and in the striatum. Scale bar: 200 μm. The inserts show double labeling of striatal neurons expressing CTIP2 (red nuclei) and CNPY2 (green cytosol). Scale bar: 20 μm. **(I,J)** Double immunostaining of CTIP2 (red) and CNPY2 (green) in the striatum. Scale bar: 200 μm.

To investigate which cells do express CNPY2 in the brain, we performed immunostainings using the anti-CNPY2 antibody and slices from the adult mouse brain. Double labeling showed that CNPY2 is present in NeuN immunopositive neurons in the brain cortex (Figures 5A–D, and the insert in Figure 5D). CNPY2 was mainly confined to neurons, as double staining using anti-GFAP, as a marker for astrocytes, showed no labeling for CNPY2 (data not shown). A detailed analysis revealed that CNPY2 was expressed by cortical neurons in layers 5/6 (Figures 5C,D). Immunostaining using anti-CNPase as a marker for oligodendrocytes was negative for CNPY2, but it marked the myelin sheath around the axons of the cortical neurons (Figures 5E,F). Cortical neurons with cell bodies in layers 5/6 make synaptic contacts with striatal neurons, constituting the corticostriatal circuitry regulating movements (Shepherd, 2013). We observed that in addition to cortical neurons, CNPY2 immunopositive cells are also present in the striatum (Figures 5A–F, and the insert in Figures 5G,H). Striatal cells are known to express CTIP2, which is a transcription factor present in medium spiny neurons (Avram et al., 2000; Arlotta et al., 2008; Desplats et al., 2008). Immunostainings confirmed that CTIP2 is expressed by the striatal neurons (Figures 5G–J), and by cortical neurons in layer 5 (Figures 5G,H). These results show that CNPY2 is present in the cortex and in the striatum of mice, and it is expressed by neurons forming the corticostriatal circuitry.

This circuitry is known to be altered in brain diseases including HD that is characterized by the loss of movement control related to the dysfunctions of striatal neurons (Eidelberg and Surmeier, 2011; Miller et al., 2011). To investigate CNPY2 expression in HD, we studied brain tissue of transgenic N171-82Q mice, as an animal model for the disease (Arlotta et al., 2008; Ramaswamy et al., 2009; Kairisalo et al., 2011; Kannike et al., 2014). Immunostaining revealed an increase in the CNPY2 immunostaining in cortical neurons in layers 5/6, as shown here for the 16 -week-old N171-82Q mice compared with age-matched controls (Figures 5A–F).

**Figure 6:**
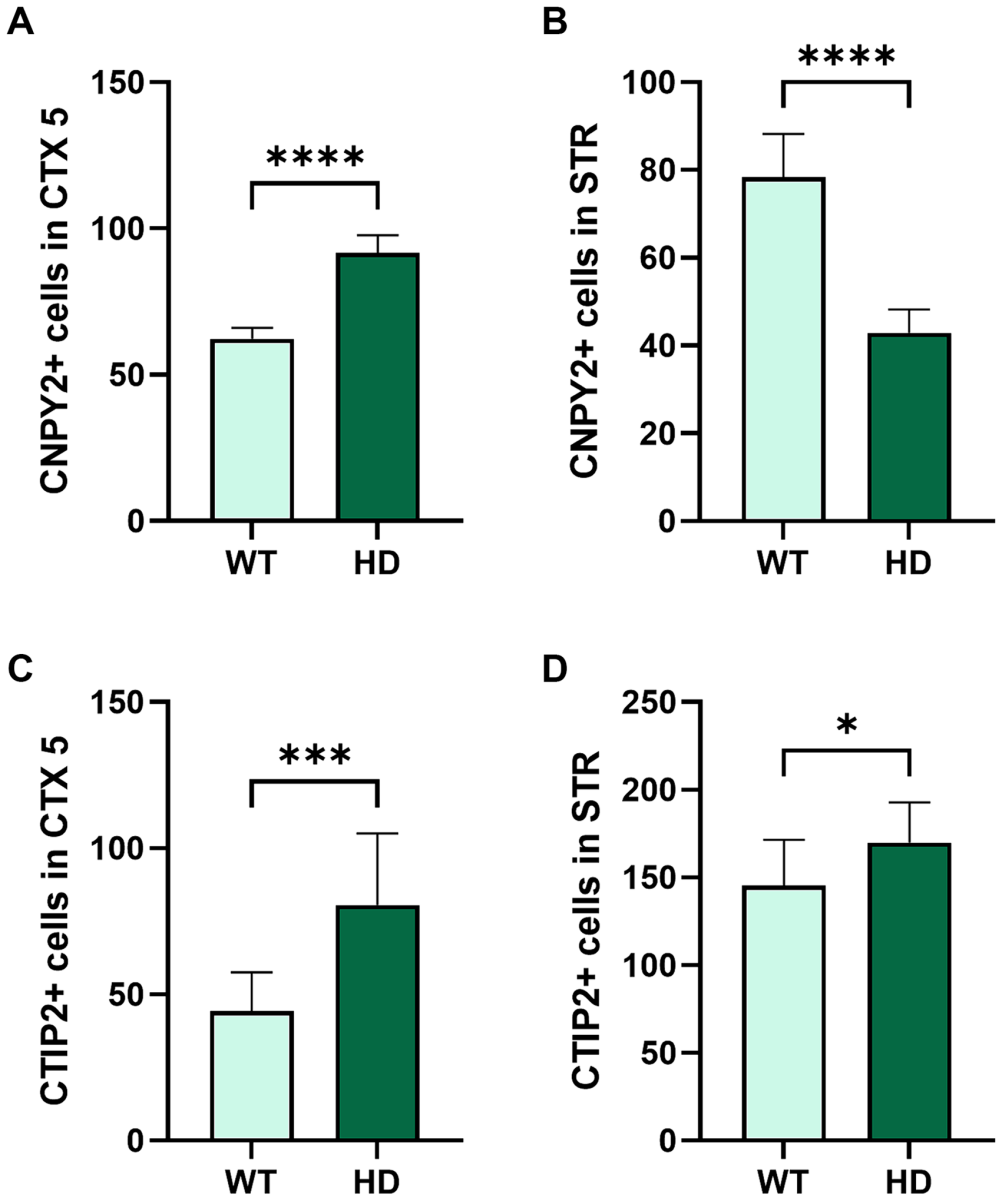
Quantification of the numbers of CNPY2 and CTIP2 immunolabeled cells in control and in N171-82Q mouse brain. The number of immunopositive CNPY2 **(A,B)** and CTIP2 cells **(C,D)** in the cortical layer 5 (CTX5) and in the striatum (STR) was determined by immunostaining in 19-week-old control mice (WT) and N171-82Q mice (HD) as described above in Figure 5. Fluorescence images were taken from three locations in CTX5 and STR, and the immunopositive cells were counted as described in methods and by using ImageJ. Free-floating slices were obtained from more than three independent control and N171-82Q mice. There was an increased number of immunopositive CNPY2 and CTIP2 cells in CTX5 **(A,C)**. In the striatum, the number of CNPY2 immunopositive cells decreased **(B)** while that of CTIP2 was slightly increased **(D)**. Values are means ± SD, *n* = 3. ^***^p < 0.001 or ^**^p < 0.01 for N171-82Q vs. control mice.

Quantification of the number of immunolabeled cells using ImageJ revealed an increase in the number of CNPY2 immunopositive cells in cortex as shown here for the layer 5 (CTX5) in the 19-week-old N171-82Q mice (Figure 6A). In contrast, the number of CNPY2 immunopositive cells in the striatum was lower in 19-week-old N171-82Q mice compared with controls (Figure 6B). We further observed that the number of immunolabeled CTIP2 neurons in CTX5 was increased in the N171-82Q mice compared with control mice (Figure 6C). In the striatum, there was also a moderate increase in the number of CTIP2 positive cells in the 19-week-old N171-82Q mice (Figure 6D). Together the results show that the number of CNPY2 immunopositive cells are increased in the cortex in the 19-week-old N171-82Q mice compared with controls. The same was also noted for the immunolabeling of CTIP2 in CTX5. In the striatum, however, there was a decrease in the immunolabeling of CNPY2 cells in 19-week-old N171-82Q mice, while that of CTIP2 was increased albeit less than in the cortex.

**Figure 7:**
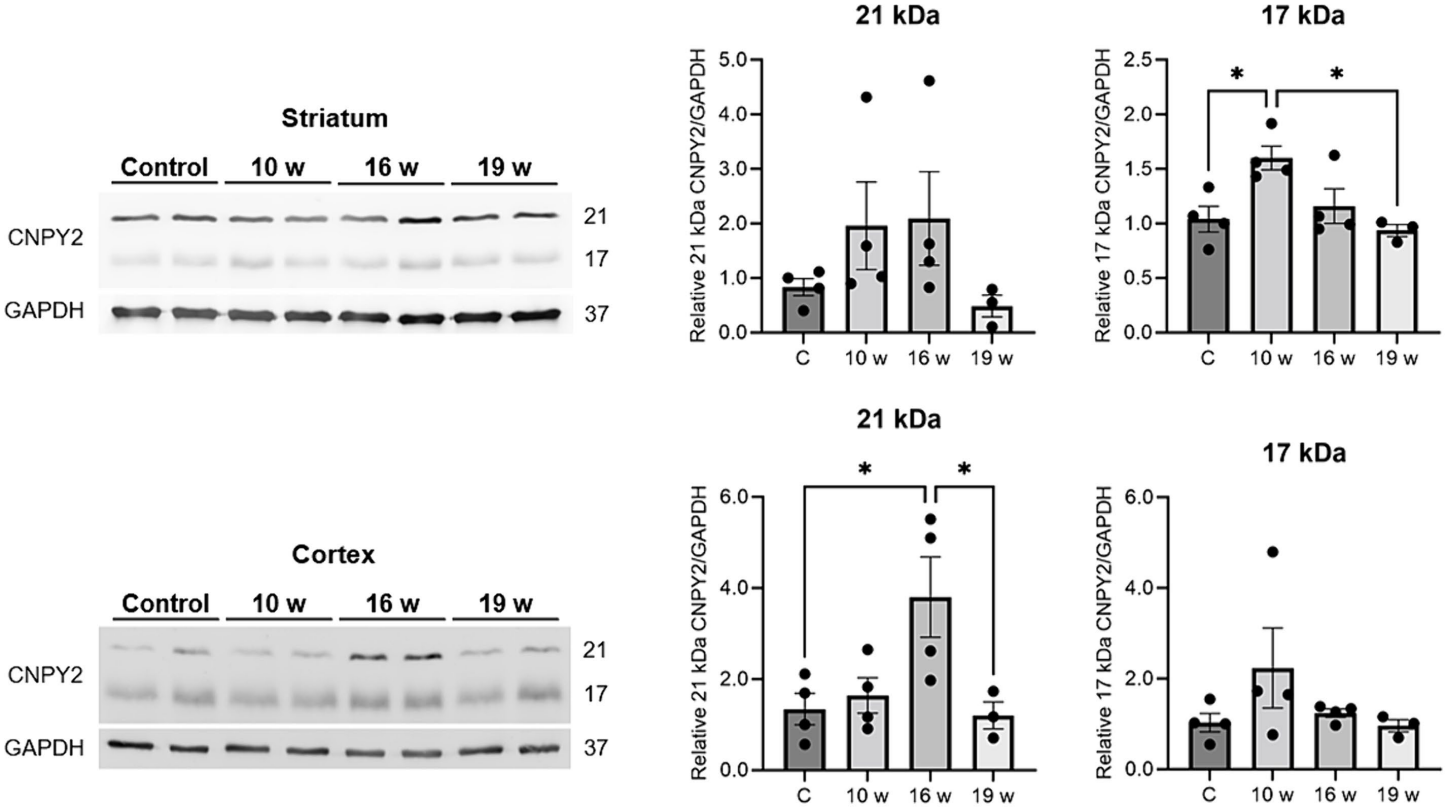
CNPY2 protein levels are altered in the N171-82Q mouse brain. Brain tissues were prepared from control and N171-82Q mice of different ages and used for immunoblotting with anti-CNPY2 antibodies as described in methods. GAPDH was used as control for the immunoblots. Left, immunoblot. Right, quantification. Upper panels, striatum. Note increases in CNPY2 in 10–16 weeks old N171-82Q mice compared with age-matched controls. Lower panels, cortex. CNPY2 was increased particularly in the 16-week-old N171-82Q mice compared with control mice. Quantification was done separately for the 21 kDa and the 17 kDa CNPY2 band. Values are means ± SD, *n* = 5. ^*^p < 0.05 for N171-82Q samples vs. control samples.

We next examined whether CNPY2 protein levels are also changed in the brain by performing immunoblots of lysates from control and N171-82Q mice. Results showed that particularly the 21 kDa band of CNPY2 was increased in the striatum of N171-82Q mice aged 10–16 weeks (Figure 7, upper panels). CNPY2 was also increased in the cortex particularly in 16-week-old N171-82Q mice (Figure 7, lower panels). In the striatum, CNPY2 levels were reduced in 19-week-old N171-82Q mice compared with age-matched controls (Figure 7, upper panels). These changes in protein expression of CNPY2 roughly correspond to the immunolabeling for CNPY2 with increases in the cortex and a late decrease in the striatum of N171-82Q mice. Unfortunately, due to the lack of materials we were not able to perform immunoblotting for CTIP2 in these experiments. Taken together these results demonstrate that CNPY2 undergoes changes in the N171-82Q mice in neurons in brain regions related to motor control.

### CNPY2 expression is protective for cultured striatal neurons

**Figure 8:**
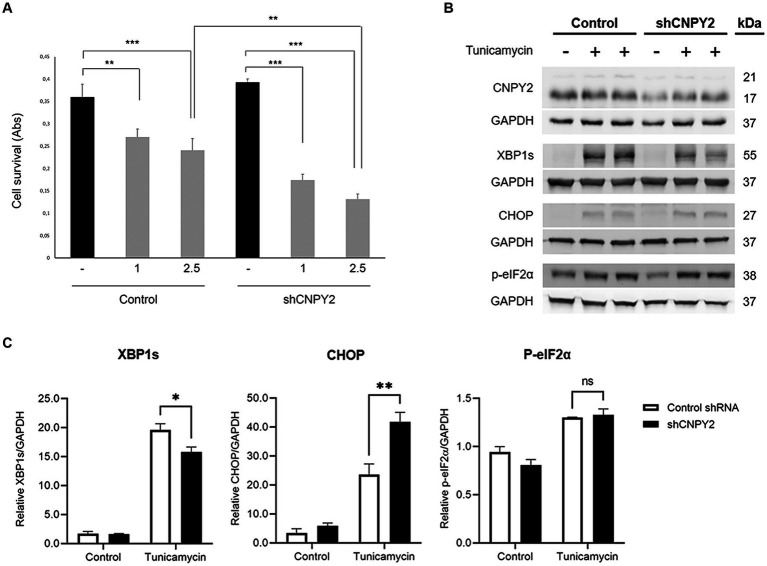
Downregulation of CNPY2 reduces the viability of cultured striatal neurons. Striatal cells were cultured in 6 well plates for immunoblotting or in 96 well plates for cell viability assays. **(A)** Striatal cells were transfected with shRNA-CNPY2 plasmid or scrambled shRNA for 24 h, and 1 or 2.5 μg/mL tunicamycin (T) was then added to half of the cells for 16 h. Cell viability was then determined using 8 wells for each treatment and the experiments were repeated. Downregulation of CNPY2 rendered the striatal cell more vulnerable to tunicamycin treatment compared with controls. Values are means ± SD, *n* = 4. ^**^p < 0.01 for 1 μg/mL T vs. C, ^***^p < 0.001 for 2.5 μg/mL vs. C in both groups, and ^**^p < 0.01 for sh-CNPY2 + 2.5 μg/mL T vs. C + 2.5 μg/mL T. **(B)** Immunoblots. Cells were treated with 2.5 μg/mL tunicamycin for 24 h. Levels of CNPY2 were reduced after shRNA-CNPY2 expression. Control cells received scrambled shRNA. Splicing of XBP1 (XBP1s) and CHOP induction were changed in CNPY2 downregulated cells. GAPDH was used as a control for blotting. **(C)** Quantification. XBP1s induced by tunicamycin was reduced after CNPY2 downregulation, whereas that of CHOP was further increased. There was no significant effect on induction of phosphorylated eIF2α level by CNPY2 downregulation. Values are means ± SD, *n* = 3. ^**^p < 0.01 or ^*^p < 0.05 for shCNPY2 + Tun vs. Tun.

The differential immunolabeling of CNPY2 in cortical and striatal neurons with age in N171-82Q mice could reflect a selective vulnerability of the latter in HD. To study the role of CNPY2 in the striatal neurons further, we downregulated CNPY2 in cultured striatal cells using shRNA. This approach reduced the relative level of CNPY2 to about half in the striatal cells (Figure 8B), which is in accordance with data obtained with mouse neuroblastoma cells (Figure 2). The addition of 1–2.5 μg/mL tunicamycin to the cells for 24 h reduced the viability of striatal cells significantly by about 30% that was increased to more than 60% after the downregulation of CNPY2 using shRNA (Figure 8A). Immunoblotting revealed that tunicamycin induced the splicing of XBP1 (XBP1s, 56 kDa band), and increased the levels of CHOP (27 kDa band), and of p-eIF2alpha (38 kDa band) in the striatal neurons (Figure 8B). Quantification of the data showed that the tunicamycin-mediated increase in XBP1s was reduced in CNPY2 downregulated cells, while that of CHOP was further elevated (Figure 8C). In these cultured striatal neurons, the ATF6 levels were too low to make any comparison between cells. Together the results demonstrate that the downregulation of CNPY2 reduced XBP1s and increased CHOP further after tunicamycin in the striatal cells. Most significantly, reducing CNPY2 levels rendered the striatal cells more vulnerable toward the action of tunicamycin with a loss of cell viability. This supports the notion that the relative level of CNPY2 is important for supporting neuronal viability and for counteracting ER stress in striatal neurons.

## Discussion

CNPY2 is an ER-localized protein expressed by different cells and tissues during development and in adult mice (Bornhauser et al., 2003; Hatta et al., 2014). Recent studies have demonstrated that CNPY2 functions in heart regeneration (Guo et al., 2015a,b; Yin et al., 2019), and in the regulation of liver metabolism associated with fatty liver (Hong et al., 2017). CNPY2 has also emerged as an important factor for the regulation of cell proliferation and viability of tumors, by modulating cancer cell signaling (Yan et al., 2016; Ito et al., 2018; Kakehashi et al., 2021).

In this work, we present evidence that CNYP2 is a trophic factor for neuronal cells by affecting UPR signaling. We observed that CNPY2 itself was increased by tunicamycin and is released from the cells. The beneficial action of CNPY2 on nerve cell viability was associated with a reduction in the levels of CHOP that is known to be upregulated during ER stress and causing cell death (Shepherd, 2013; Li et al., 2015). CNPY2 was further linked to higher levels of the transcriptionally active 56 kDa fragment of ATF6α indicating an effect of CNPY2 on this branch of the UPR. In contrast, we did not observe a significant change in the tunicamycin-mediated activation/phosphorylation of eIF2α by CNPY2. Activation of PERK causes eIF2α phosphorylation and translational block during the UPR, but eIF2α is also phosphorylated by other cellular kinases related to cell stress responses. We were unable to directly study the pPERK levels in our cell model due to the lack of specificity of the commonly used anti-PERK antibodies (Stone et al., 2019). To investigate IRE1α in the neuronal cells, we examined the levels of spliced (XBP1s) that were increased by CNPY2 overexpression, whereas downregulation of CNPY2 by shRNA decreased XBP1s levels in striatal neurons. These experiments demonstrate that CNPY2 affects IRE1α and ATF6 signaling pathways in neurons, and is itself upregulated by UPR, suggesting a feed-forward loop to control ER stress. Recently it was reported that increases in CNPY2 expression are linked to an activation of the PERK pathway during UPR stress in cultured hepatocytes (Hong et al., 2017). This evidence together with the present findings show that CNPY2 is regulated by the UPR although the precise mechanisms may vary between different cell types such as neurons and hepatocytes.

In this study, we also observed that CNPY2 is released from neuronal cells into the culture medium in line with an autocrine or paracrine mode of action. Moreover, the addition of recombinant CNPY2 increased the viability of cortical neurons challenged with tunicamycin for 24 h. This indicates that CNPY2 can also act on neurons from outside of the cell. In accordance with this, it has been shown that CNPY2 influences smooth muscle cells in the context of heart injury (Guo et al., 2015b). In CNPY2 there is the HDEL sequence at the carboxyterminal end (Bornhauser et al., 2003), which resembles the KDEL sequence mediating binding to the KDEL receptors in the ER. CNPY2 was further demonstrated to interact with the ER chaperone BiP/Grp78 in hepatocytes under conditions of ER stress (Hong et al., 2017). CNPY2 can be compared with mesencephalic astrocyte-derived growth factor (MANF) another ER-linked protein that binds BiP/Grp78 and is released from cells into the culture medium (Glembotski et al., 2012). Taken together our results indicate that CNPY2 can be secreted from neuronal cells. However, the mechanisms by which CNPY2 can act on cells from outside warrant further investigations.

Previously, it was shown that CNPY2 is present in different brain areas including the hippocampus (Bornhauser et al., 2003; Hatta et al., 2014). Herein we focused on the cortex and the striatum and observed that cortical neurons in the layers 2/3 and layers 5/6 are immunopositive for CNPY2. Striatal neurons that receive inputs from the cortical neurons were also immunoreactive for CNPY2. These neurons are part of the corticostriatal circuitry that is important in the control of movements (Eidelberg and Surmeier, 2011; Miller et al., 2011; Shepherd, 2013). In HD, the striatal neurons are vulnerable during the course of the disease but the role of the projecting cortical neurons to the striatal pathology is not fully understood. Studies using electrophysiology *in vivo* in HD models as well as brain imaging, have revealed changes in the neural activity pattern in the corticostriatal network (Eidelberg and Surmeier, 2011; Miller et al., 2011). Results obtained in this study showed that the number of CNPY2-immunopositive cells was upregulated in the cortex, but downregulated in the striatum in 19-week-old N171-82Q mice. The decrease in CNPY2-immunopositive cells in the striatum is associated in time with the progression of the disease pathology in this mouse model (Ferrante, 2009; Ramaswamy et al., 2009; Stepanova et al., 2023).

Double labeling experiments revealed that CNPY2 is co-expressed with the protein CTIP2 in striatal and in some cortical neurons CTIP2 is a transcription factor, also named B-cell lymphoma/leukemia 11 B (BCL11B) affecting the development and survival of T lymphocytes (Avram et al., 2000). CTIP2 acts as a tumor suppressor and plays a role in blood cancer such as leukemias (Avram et al., 2000). In the brain, CTIP2 is expressed by neurons in the striatum and in the cortex mainly in layer 5 (Arlotta et al., 2008; Desplats et al., 2008), and the protein functions in the differentiation of striatal neurons during development (Arlotta et al., 2008). As a transcription factor, CTIP2 acts by influencing the expression of genes some of which have also relevance for brain disorders, such as HD (Desplats et al., 2008; Ahmed et al., 2015; Song et al., 2022). Further, it was shown that CTIP2 is reduced in the HD R6/1 transgenic mouse model and in postmortem samples of the striatum from patients with HD (Desplats et al., 2008; Song et al., 2022). In this work, we observed that the number of CTIP2 immunolabeled cells was increased in the cortex of 19-week-old N171-82Q mice and stayed largely constant in the striatum compared with age-matched controls. It is possible that the levels of CTIP2 will be reduced during a later stage in the N171-82Q mice. More studies on CTIP2 and its relationship to CNPY2 using aged mice are warranted to resolve this question.

There are different types of neurons in the cortex, including intra-telencephalic (IT) and pyramidal tract (PT) type neurons having their cell bodies in layers 2/3 and layer 5, respectively (Shepherd, 2013). CNPY2 was expressed by cortical neurons in both of these layers, but CTIP2 was mainly expressed by the PT neurons, in addition to the medium spiny neurons. In the 19-week-old N171-82Q mice, the number of CNPY2 immunopositive neurons was reduced in the striatum, but increased in the cortex. This may contribute to the enhanced viability of cortical neurons, as compared with striatal cells in the HD N171-82Q mice. Along with this, we observed that the addition of recombinant CNPY2 was able to protect cortical neurons in culture against ER stress induced by 24 h of tunicamycin. To investigate the role of CNPY2 in striatal neurons, we employed cells from the mouse striatum with the expression of normal 7Q Htt protein (Zuccato et al., 2001). Data revealed that downregulation of CNPY2 using shRNA decreased the cell viability of these neurons after tunicamycin as compared with controls. The enhanced cell death was further related to an increase in CHOP levels after CNPY2 reduction. The mechanisms by which CNPY2 act on ER stress are summarized in the Graphical Abstract, showing a reduction in CHOP and in the ATF6 pathway. The PERK- eIF2α pathway was not significantly influenced by CNPY2 neurons but has been linked with CNPY2 in hepatocytes (Hong et al., 2017). As shown here, the IRE1α pathway and the transcription factor XBP1s were increased by CNPY2. Further studies will focus on the characterization of downstream genes regulated by CNPY2 in neurons. A summary of our study is given in the Graphical Abstract that depicts the signaling pathways for UPR/ER stress and how these can be affected by CNPY2.

In conclusion, the results lend credence to the view that CNPY2 acts as a neurotrophic factor for neuronal cells including striatal and cortical neurons by influencing ER stress signaling. CNPY2 itself is increased by the UPR and is elevated in the N171-82Q mouse brain tissue reflecting an adaptive UPR. Immunoblotting revealed that there was an early increase in CNPY2 in the N171-82Q mouse brain followed by a reduction during later stages. CNPY2 is co-expressed with the transcription factor CTIP2 in cortical and striatal neurons suggesting a possible functional relationship. At 19–20 weeks, the number of CNPY2 positive neurons decreased in the striatum in the N171-82Q mouse brain concomitant with the worsening of HD pathology. These results demonstrate that CNPY2 is altered in the N171-82Q mice and is expressed by corticostriatal neurons involved in the regulation of movements. It will be important in the future to study the roles of CNPY2 *in vivo* using gene deleted animals and other models of neurological diseases.

## Data Availability

The raw data supporting the conclusions of this article will be made available by the authors, without undue reservation.
