# Supplementary material for: CNPY2 protects against ER stress and is expressed by corticostriatal neurons together with CTIP2 in a mouse model of Huntington’s disease
Source: Front Mol Neurosci. 2024 Sep 18;17:1473058. doi: 10.3389/fnmol.2024.1473058 (PMC11446244; doi:10.3389/fnmol.2024.1473058)
Supplement: Supplementary file 1 [file Data_Sheet_1.PDF]

Figure 1:

For Figure 1A

Two experiments are shown in the original blot. Note, CNPY2 was named MSAP previously**.**

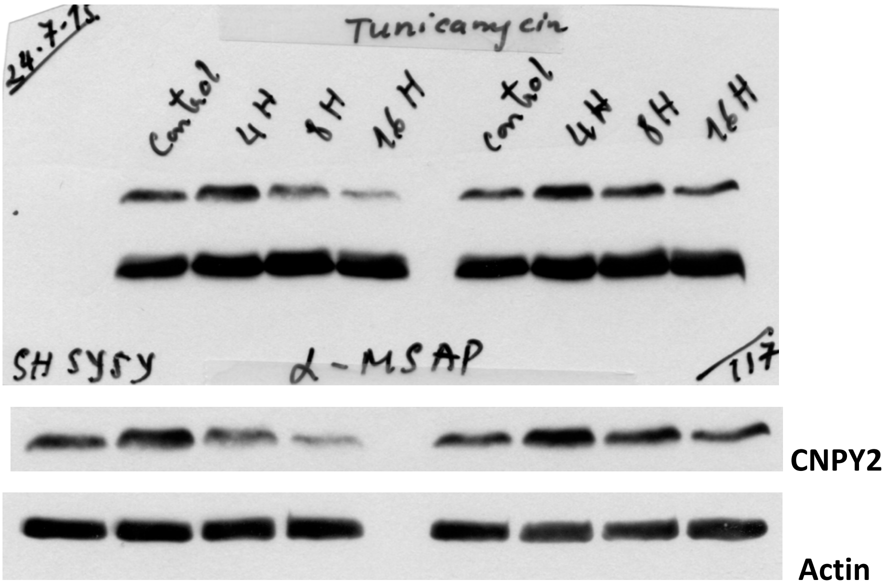

Note in this blot above the Molecular weight bands were not marked and the whole blot was not found anymore.

Below then, is another film of a blot done around the same time using Tunicamyicn (Tun) and showing the whole blot with the Molecular bands marked.

CNPY2 is at **around 21 and 17 kDa**. There are unspecific bands above at 36kDa and at 55 kDa in the longer exposure as shown here.

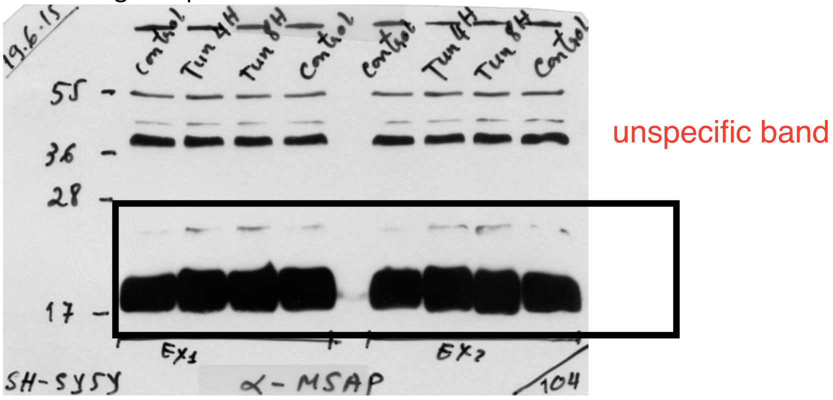

For Figure 1 D. Whole original blots are already shown in the Ms in Figure 1D.

Figure 2:

Figure 2B. Downregulation of CNPY2 using shRNA shown are based upon experiments in triplicates. Original blot is below. CNPY2 is at 17kDa with this exposure time. Unspecific band at 36kDa.

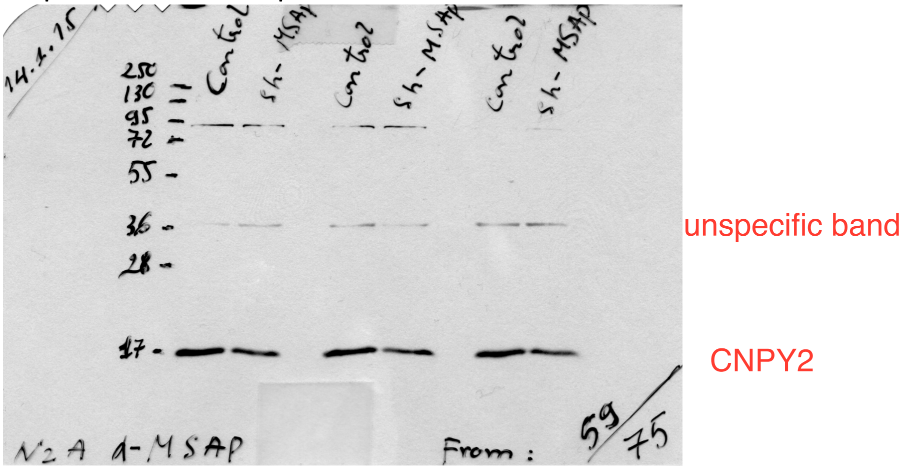

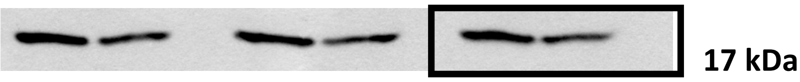

3A

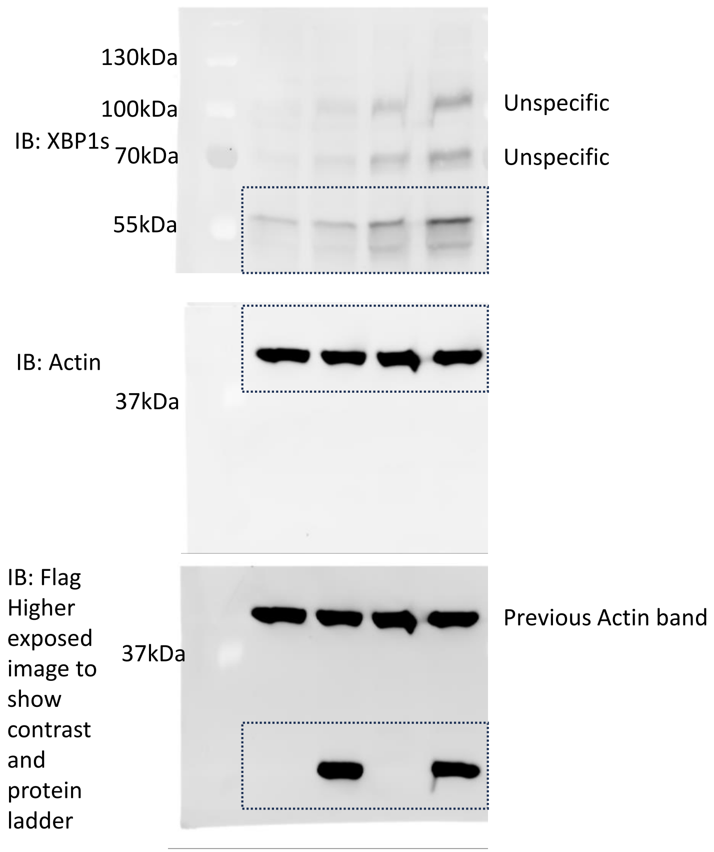
130kDa
100kDa
70kDa
55kDa
IB: XBP1s
Unspecific
Unspecific
IB: Actin
37kDa
IB: Flag
Higher exposed image to show contrast and protein ladder
37kDa
Previous Actin band

3B

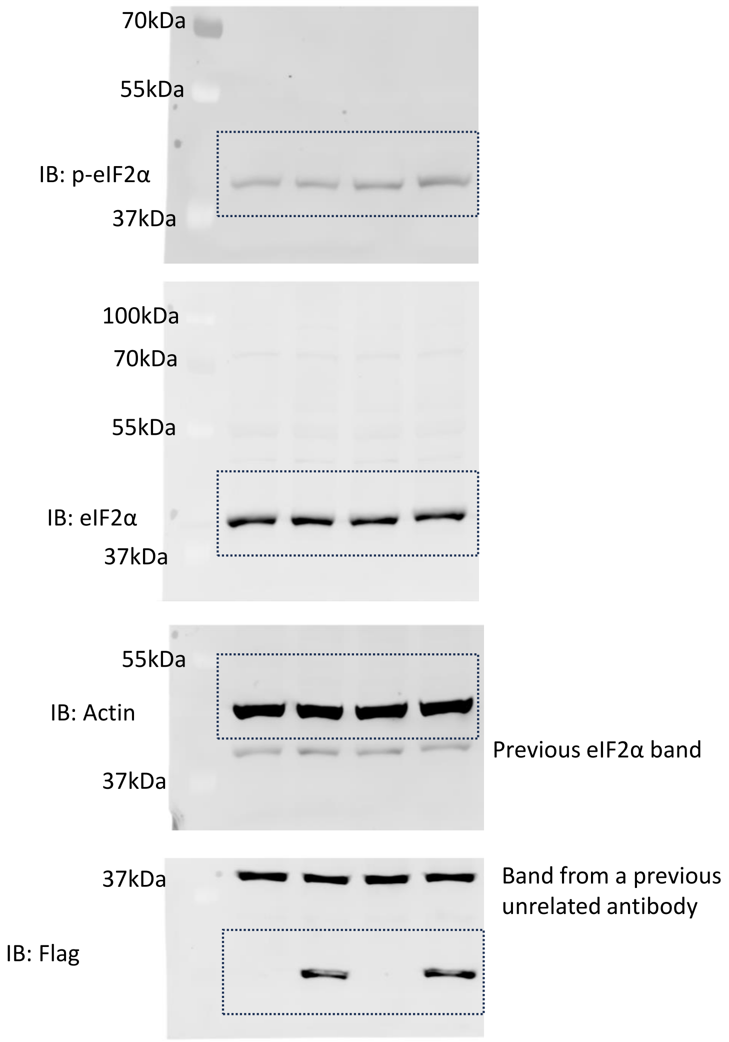
70kDa
55kDa
IB: p-eIF2α
37kDa
100kDa
70kDa
55kDa
IB: eIF2α
37kDa
55kDa
IB: Actin
37kDa
Previous eIF2α band
37kDa
IB: Flag
Band from a previous unrelated antibody

Figure 3C-D:

Figure 3C. Original blots of changes in **ATF6 processing** after expression of CNPY2=MSAP. Here are two experiments, left side, right side with 2 exposures times shown, upper and lower. The right one experiment is shown **as Figure 3C**.

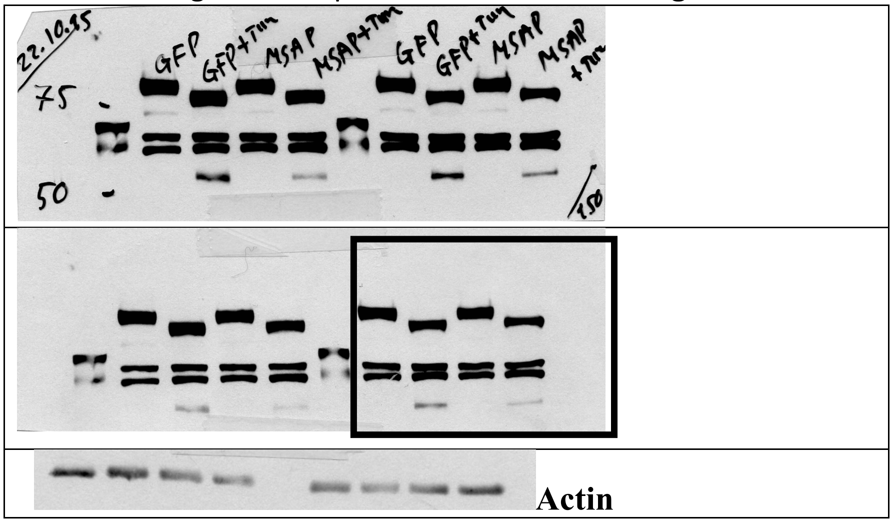

Actin

Figure 3D.**Original Blots of changes in CHOP (indicated by arrow)** after expression of CNPY2=MSAP. Here are two experiments, left side, right side with 2 exposures times shown, upper and lower. The specific band of CHOP is at 27kDa (arrow). The right one experiment is shown **in Figure 3D**.

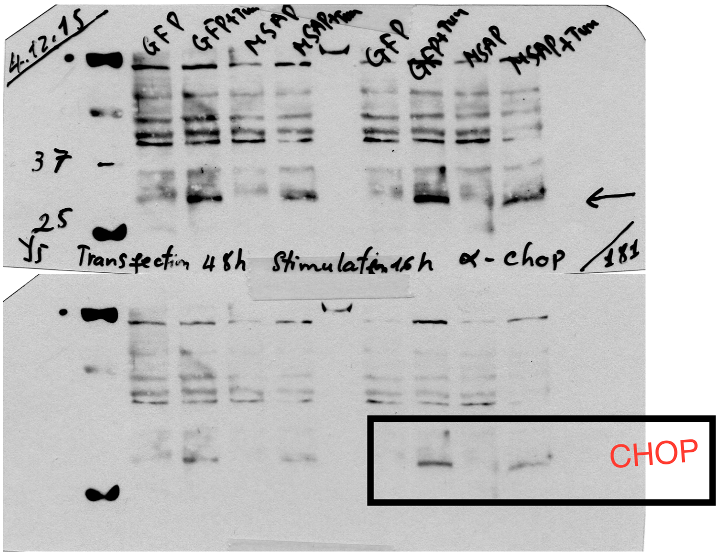

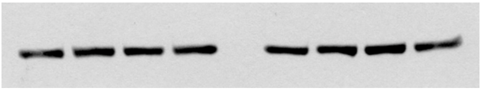

Actin

FIGURE 7. CNPY2 protein expression in Control and HD mice

Upper blots Striatum,

Lanes 1-2 Controls; 3-4, 10w HD: 5-6, 16weeks HD, 7-9 19 weeks HD. CNPY2 is at 21 kDa and 17kDa. There is a week unspecific band at 35kDa using this antibody.

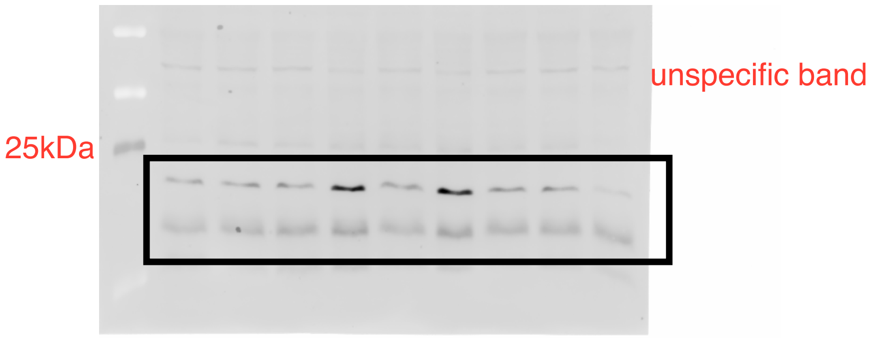

CNPY2

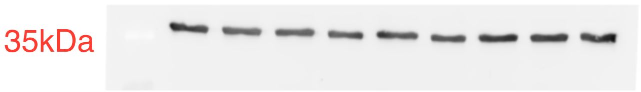

GAPHD

Lower blots Cortex

Note the lanes are reversed from left of the markers; Lanes 1-2 Controls; 3-4, 10w HD: 5-6, 16weeks HD, 7-9 19 weeks HD,
The same information as above, CNPY2 at 21kDa and 17 kDa bands.

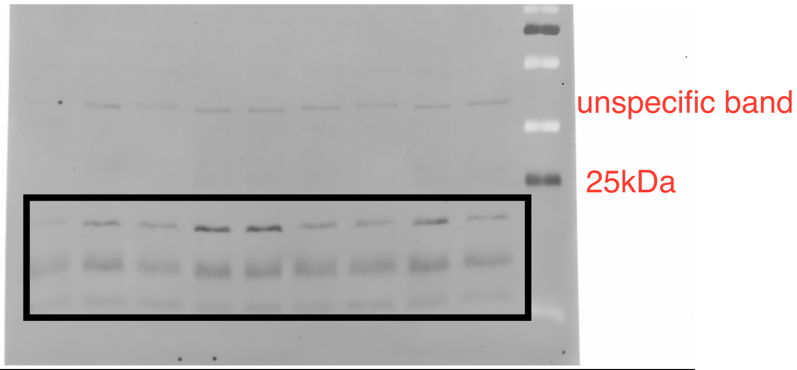

CNPY2

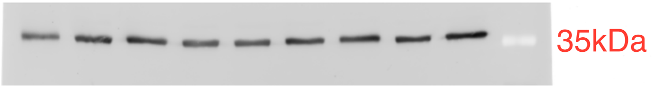

GAPDH

FIGURE 8. **CNPY2 downregulation and effects of ER stress in striatal neurons**

Figure 8D. uppermost is a **CNPY2 blot**: lanes 1-3 shRNA-control; lanes 4-6 shRNA-CNPY2. **CNPY2** is at around 21kDa and 17kDa.

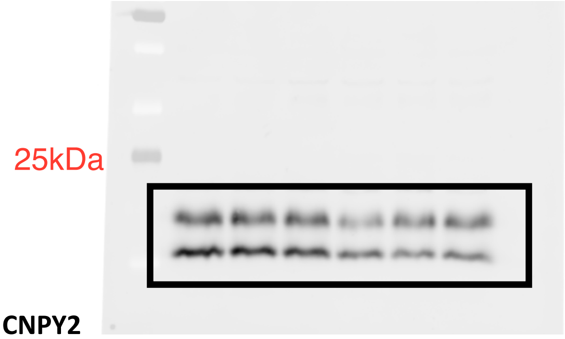

CNPY2

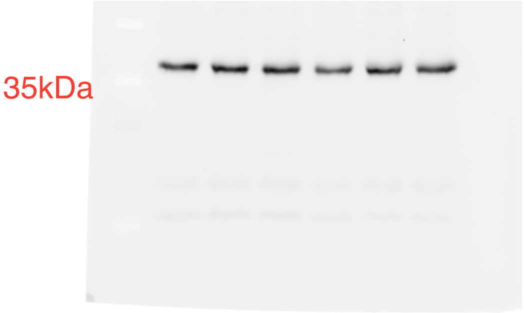

GAPHD

XBP1 blot: **spliced form of XBP1** is at 55 kDa band, The lower band here at **37kDa shows GAPDH control** done before.
Description: lanes 1-3 shRNA-control; lanes 4-6 shRNA-CNPY2.Lane 1 and 4 untreated controls, Lanes 2,3 and 5,6 treatment with Tunicamycin,

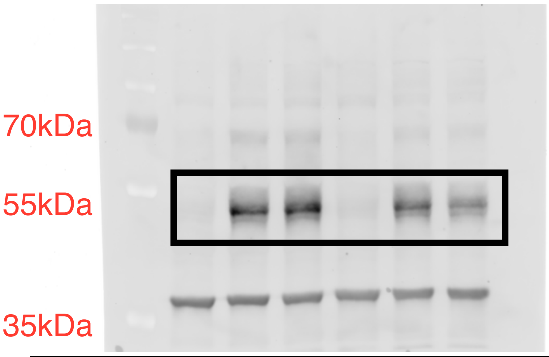

XBP1s

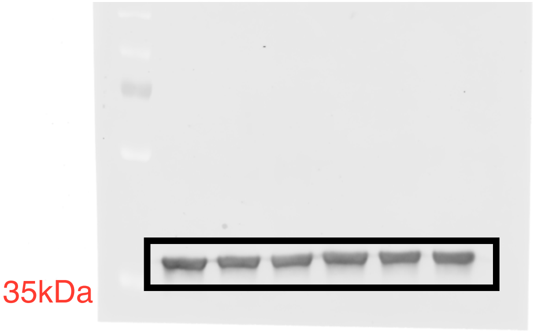

GAPDH

CHOP blot: Description as above but note that the order of the lanes are in reverse order from left of the marker: lanes 1-3 control, lanes 4-6 CNP2 downregulation. CHOP is the band at 27 kDa. Note unspecific band at around 32 kDa below the 35kDa marker. GAPDH is 37kDa.

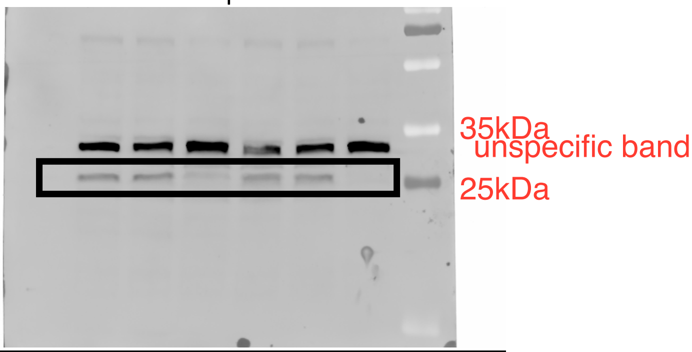

CHOP

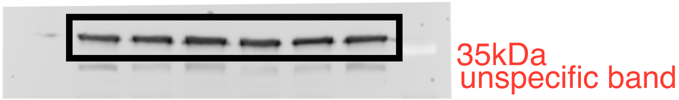

GAPDH

p-eIF2alpha blot: Description as above but note that in the upper blot the Lanes are in reverse order-from left of the marker 1-3, controls; lanes 4-6 CNPY2 downregulation. P-eIF2 is shown at 38kDa band a nice antibody!
Lower blot with right order from the marker, lanes 1-3 control; lanes 4-6, CNPY2 downregulation. GAPDH is a control shown at 37 kDa and done afterwards.

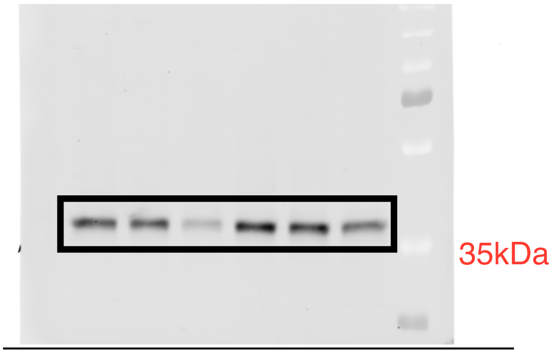

eIF2alpha

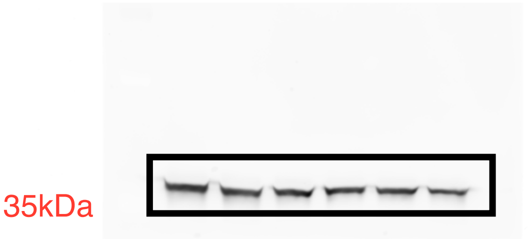

GAPDH
