# Supplementary figures and images for: CNPY2 protects against ER stress and is expressed by corticostriatal neurons together with CTIP2 in a mouse model of Huntington’s disease
Source: Front Mol Neurosci. 2024 Sep 18;17:1473058. doi: 10.3389/fnmol.2024.1473058 (PMC11446244; doi:10.3389/fnmol.2024.1473058)

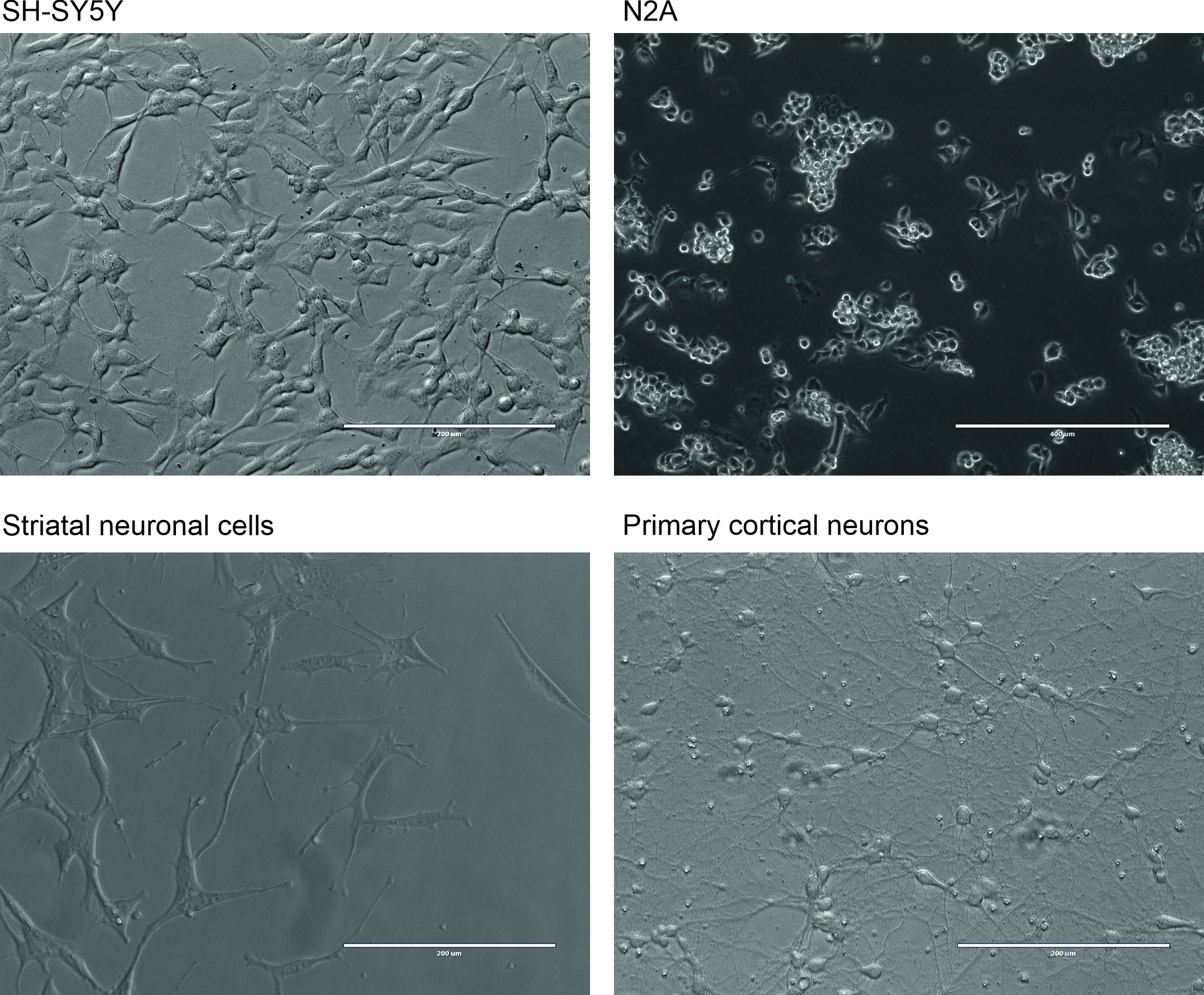

Supplement: Supplementary file 2 [file Image_1.TIF]

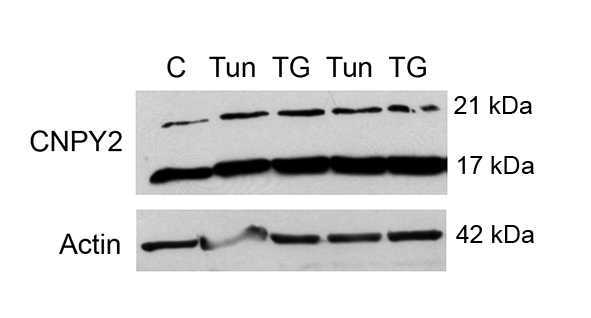

Supplement: Supplementary file 3 [file Image_2.TIF]

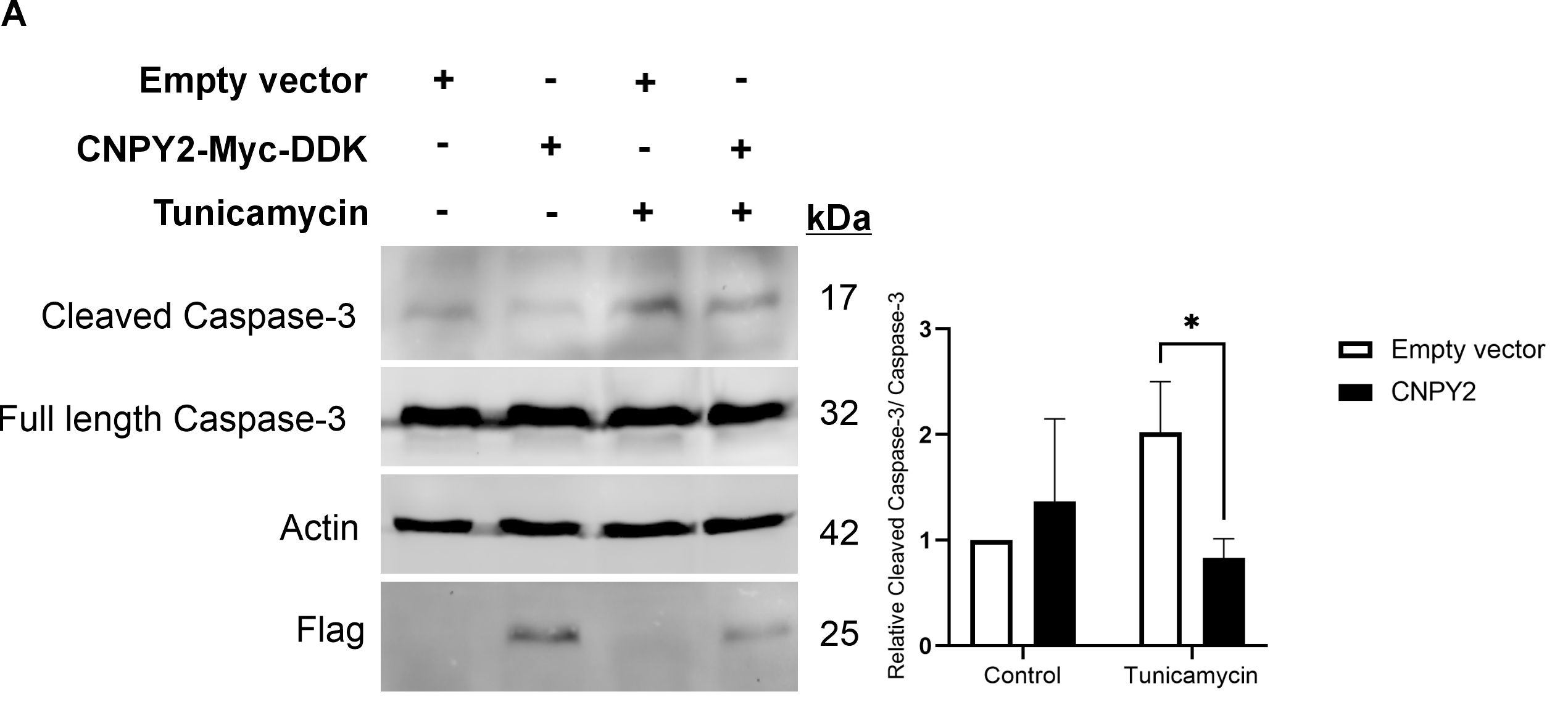

Supplement: Supplementary file 4 [file Image_3.TIF]

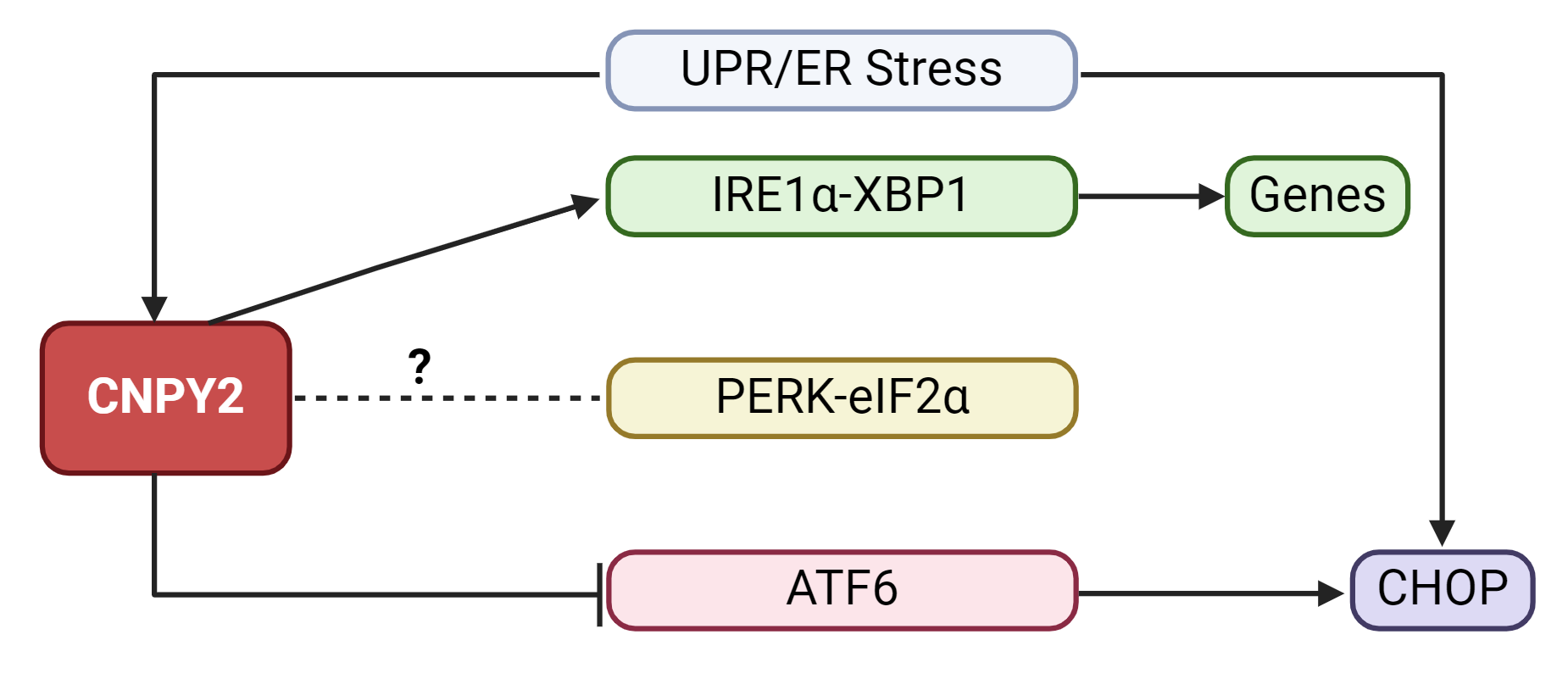

Supplement: Supplementary file 5 [file Image_4.PNG]
