# Supplementary material for: CNPY2 protects against ER stress and is expressed by corticostriatal neurons together with CTIP2 in a mouse model of Huntington’s disease
Source: Front Mol Neurosci. 2024 Sep 18;17:1473058. doi: 10.3389/fnmol.2024.1473058 (PMC11446244; doi:10.3389/fnmol.2024.1473058)
Supplement: Supplementary file 6 [file Table_1.DOCX]

**Supplementary Table.**

**Summary of number of animals used for immunostaining**

| **Age** | **Genotyp** | **Gender** | **Number** |
| --- | --- | --- | --- |
| *10 weeks* | N71-82Q | female | 2 |
|  |  | male | 2 |
| 16 weeks | N71-82Q | female | 11 |
|  |  | male | 13 |
| 19-20 weeks | N71-82Q | female | 11 |
|  |  | male | 3 |
| *10weeks* | Wildtype | female | 2 |
| 16 weeks | Wildtype | female | 4 |
|  |  | male | 7 |
| 20 weeks | Wildtype | female | 6 |
|  |  | male | 6 |
